# GFAT1-linked TAB1 glutamylation sustains p38 MAPK activation and promotes lung cancer cell survival under glucose starvation

**DOI:** 10.1038/s41421-022-00423-0

**Published:** 2022-08-09

**Authors:** Shupei Wei, Qin Zhao, Ke Zheng, Peiying Liu, Nannan Sha, Yingzi Li, Chunmin Ma, Jingjie Li, Lingang Zhuo, Guanxin Liu, Wenhua Liang, Yuhui Jiang, Tao Chen, Nanshan Zhong

**Affiliations:** 1 State Key Laboratory of Respiratory Disease; National Clinical Research Center of Respiratory Disease; Guangzhou Institute of Respiratory Health, First Affiliated Hospital of Guangzhou Medical University, Guangzhou, Guangdong China; 2 Key Laboratory for Cell Homeostasis and Cancer Research of Guangdong Higher Education Institutes, Affiliated Cancer Hospital and Institute of Guangzhou Medical University, Guangzhou, Guangdong China; 3 Department of Liver Surgery and Shanghai Cancer Institute, State Key Laboratory of Oncogenes and Related Genes, Shanghai Cancer Institute, Ren Ji Hospital, Shanghai Jiao Tong University School of Medicine, Shanghai, China; 4 Yangjiang Key Laboratory of Respiratory Disease, Department of Respiratory Medicine, People’s Hospital of Yangjiang, Yangjiang, Guangdong China

**Keywords:** Cancer metabolism, Non-small-cell lung cancer

## Abstract

Reprogrammed cell metabolism is deemed as one of the hallmarks of cancer. Hexosamine biosynthesis pathway (HBP) acts as an “energy sensor” in cells to regulate metabolic fluxes. Glutamine-fructose-6-phosphate amidotransferase 1 (GFAT1), the rate-limiting enzyme of HBP, is broadly found with elevated expression in human cancers though its exact and concrete role in tumorigenesis still remains unknown and needs further investigation. P38 mitogen-activated protein kinase (MAPK) is an important component of stress-signaling pathway and plays a critical role in cell fate decision, whereas the underlying mechanism of its activation under nutrient stress also remains elusive. In this study, we show that glucose deprivation induces the interaction of GFAT1 with transforming growth factor β-activated kinase 1 binding protein 1 (TAB1) in a TAB1 S438 phosphorylation-dependent manner. Subsequently, the binding of GFAT1 to TAB1 facilitates TTLL5–GFAT1–TAB1 complex formation, and the metabolic activity of GFAT1 for glutamate production further contributes to TTLL5-mediated TAB1 glutamylation. In consequence, TAB1 glutamylation promotes the recruitment of p38α MAPK and thus drives p38 MAPK activation. Physiologically, GFAT1-TAB1-p38 signaling promotes autophagy occurrence and thus protects tumor cell survival under glucose deficiency. Clinical analysis indicates that both GFAT1 and TAB1 S438 phosphorylation levels correlate with the poor prognosis of lung adenocarcinoma patients. These findings altogether uncover an unidentified mechanism underlying p38 MAPK signaling regulation by metabolic enzyme upon nutrient stress and provide theoretical rationality of targeting GFAT1 for cancer treatment.

## Introduction

Reprogramed metabolism provides energy and biomacromolecules to support unlimited proliferation of cancer cells, which is executed by abnormally expressed metabolic enzymes^1^. Meanwhile, these enzymes could play a crucial non-metabolic function in cancer progression by regulating signal transduction, especially under nutritional stress^2^.

The hexosamine biosynthesis pathway (HBP) is an important branch of glucose metabolism that makes use of fructose-6-phosphate, glutamine, acetyl-CoA and UTP to synthesize the end-product uridine diphosphate-N-acetylglucosamine (UDP-GlcNAc), which integrates glucose metabolism, glutaminolysis, fatty acid metabolism, and nucleotide metabolism and acts as an intracellular energy sensor. Enhanced HBP occurs in various types of cancer^3,4^.

The rate-limiting enzyme of HBP is glutamine-fructose-6-phosphate amidotransferase 1 (GFAT1), which has been found overexpressed in different types of cancer and correlates with poor prognosis^5,6^. Previous studies indicate that GFAT1 is upregulated under glucose deprivation and HBP is enhanced to support cell survival^7,8^. However, whether GFAT1 carries non-metabolic function when it is upregulated to response to nutritional stress is unclear.

Glutamylation, a modification that adds glutamate onto γ-carboxyl groups of glutamic acid residues in targeted protein, is first found on tubulin^9^, which is catalyzed by polyglutamylases from tubulin tyrosine ligase-like (TTLL) family and disaggregated by cytosolic carboxypeptidases (CCPs) family^10–13^. In addition to tubulin, glutamylation has been found on many proteins and modulate cellular activities, for instance, glutamylation of cGAS impedes its DNA-binding ability and synthase activity^14^, and glutamylation of Klf4 competes with ubiquitination and sustains protein stability during cell reprogramming^15^.

p38 MAPK signaling is a stress sensor that responds to diverse stimuli, including DNA damage, growth factors, starvation, etc^16^. Emerging evidence suggests a dual role of p38 MAPK in cancer development under distinct contexts and circumstances. The negative effect of p38 MAPK signaling in tumorignesis was initially revealed by the studies showing its inhibitory effects on RAS-induced cell transformation and cell cycle progression^17^, and suggested by the detection of changed expression of upstream regulators with repressed p38 MAPK signaling in human tumors^18,19^. In contrast, the enhanced p38 MAPK phosphorylation level was also found to be associated with malignancy in distinct types of cancers such as lung, breast carcinomas, and head and neck squamous cell carcinomas^20–22^.

p38 MAPK is canonically phosphorylated and activated by its upstream kinases MKKs upon environmental and genotoxic stress^23^. Alternatively, p38α MAPK also undergoes autophosphorylation by interacting with transforming growth factor β-activated kinase 1 binding protein 1 (TAB1), which leads to subsequent conformation alteration of p38α^24,25^. During myocardial ischemia, p38α autophosphorylation promotes glucose uptake and GLUT4 translocation^26^. In this regard, the regulatory mechanism underlying p38α autophosphorylation and its potential implication in cancer development warrants further investigation.

In this study, we reveal that glucose deficiency induces JNK/p38 MAPK-dependent interaction between GFAT1 and TAB1, by which the metabolic activity of GFAT1 drives TAB1 glutamylation in a TTLL5-dependent manner. Glutamylation of TAB1 promotes its recruitment to p38α MAPK, which facilitates p38α MAPK autophosphorylation that is critical for the downstream events that support cancer cells survival under glucose deficiency.

## Results

### GFAT1 promotes p38 MAPK activation and cell survival under glucose deficiency

**Fig. 1:**
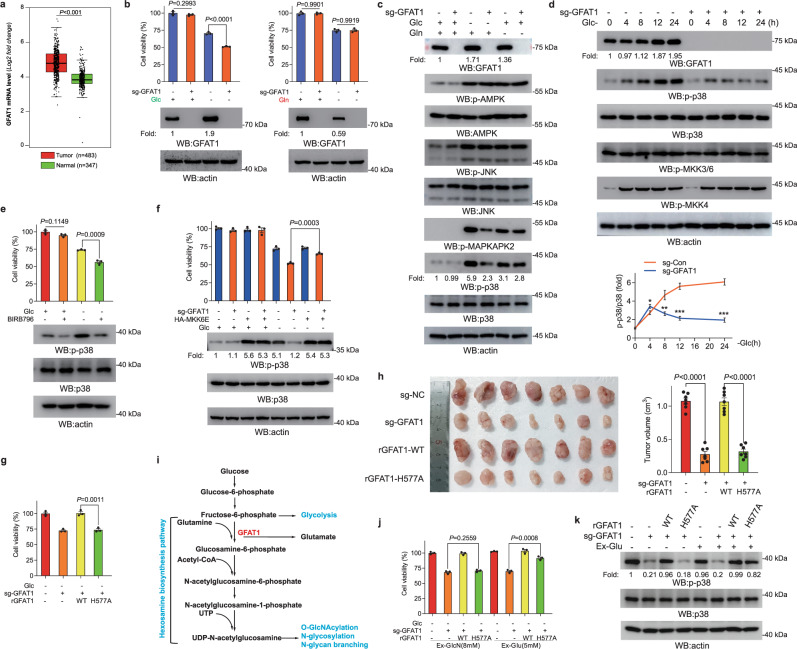
GFAT1 activates p38 MAPK and promotes cell survival and tumorigenesis through its enzymatic activity. **a** The expression of GFAT1 in lung adenocarcinoma tissues based on GEPIA. **b** Glucose deprivation, but not glutamine deprivation, inhibited cell survival in GFAT1-deleted cells. A549 cells with or without deleted GFAT1 were cultured for 36 h under glucose or glutamine deprivation. Immunoblotting analyses were performed using the indicated antibodies. Cellular viability was examined by CCK8 assay. **c** GFAT1 deletion inhibited p38 activation. A549 cells with or without deleted GFAT1 were cultured for 12 h under glucose or glutamine deprivation. Immunoblotting analyses were performed using the indicated antibodies. **d** GFAT1 deletion inhibited p38 activation at late time points. A549 cells with or without deleted GFAT1 were cultured for indicated time under glucose deprivation. Immunoblotting analyses were performed using the indicated antibodies. **e** p38 suppression impaired cell survival. A549 cells were pretreated with or without BIRB796 (1 μM) for 1 h before being cultured under glucose deprivation. Immunoblotting analyses were performed using the indicated antibodies. Cellular viability was examined by CCK8 assay at 36 h post glucose deprivation treatment. **f** Expression of MKK6E rescued cell survival in GFAT1-deleted cells. A549 cells with or without deleted GFAT1 and expression of HA-MKK6E were cultured under glucose deprivation. Cellular viability was examined by CCK8 assay at 36 h post glucose deprivation treatment. Immunoblotting analyses were performed using the indicated antibodies. **g** Expression of rGFAT1-H577A inhibited cell survival. A549 cells with or without deleted GFAT1 and reconstituted expression of WT rGFAT1 or rGFAT1-H577A were cultured under glucose deprivation. Cellular viability was examined by CCK8 assay at 36 h post glucose deprivation treatment. **h** GFAT1 deletion or expression of rGFAT1-H577A inhibited tumorigenesis. A total of 5 × 10^6^ A549 cells with GFAT1 deletion and/or reconstituted expression of the WT rGFAT1 or rGFAT1-H577A were subcutaneously injected into athymic nude mice. Representative tumor xenografts are shown. Tumor volumes were measured by using length “a” and width “b” and calculated using the following equation: V = ab^2^/2. **i** A schematic diagram of the HBP. **j** Exogenous glutamate rescued cell survival in rGFAT1-H577A-expressing cells. A549 cells with or without deleted GFAT1 and reconstituted expression of WT rGFAT1 or rGFAT1-H577A were cultured under glucose deprivation and were added with indicated concentration of exogenous Glucosamine (Ex-GlcN) or glutamate (Ex-Glu). **k** Exogenous glutamate rescued p38 activation in rGFAT1-H577A-expressing cells. A549 cells with or without deleted GFAT1 and reconstituted expression of WT rGFAT1 or rGFAT1-H577A were cultured for 8 h under glucose deprivation and were added with 5 mM exogenous glutamate (Ex-Glu). Immunoblotting analyses were performed using the indicated antibodies. In **a**, **b**, **d**, **e**, **h** and **j**, the values are presented as means ± SEM, *n* = 3; *P* values (*P < 0.05, **P < 0.01 ***P < 0.001, Student’s *t*-test, two-sided) with control or the indicated groups are presented.

The gene Expression Profiling Interactive Analysis (GEPIA)^27^ of the Cancer Genome Atlas (TCGA) and Genotype-Tissue Expression Program (GTEx) databases indicated that GFAT1 was largely upregulated in lung adenocarcinoma cancer specimens compared to adjacent normal tissue (Fig. 1a). To determine the concrete function of GFAT1 in tumorigenesis of lung adenocarcinoma, the effects of GFAT1 deletion were first examined in lung adenocarcinoma cell lines A549 and H1299 under nutrient starvation condition. GFAT1 deletion by CRISPR-Cas9 system resulted in dramatic decrease of cell survival under glucose deprivation instead of glutamine deprivation (Fig. 1b; Supplementary Fig. S1a), which is in line with the observation that the expression of GFAT1 was elevated during prolonged glucose deprivation while diminished under extended glutamine deprivation (Supplementary Fig. S1b, c). To test whether this physiological effect was linked to the canonical responsive signaling upon glucose deprivation, we examined the activation status of AMPK, p38 MAPK, and JNK. Intriguingly, GFAT1 deletion exclusively suppressed glucose deprivation-induced p38 MAPK activation, as indicated by p38 MAPK Thr180/Tyr182 phosphorylation and the downstream MAPKAPK2 Thr222 phosphorylation (Fig. 1c). Time-course analysis indicated that the negative effect of GFAT1 deletion on p38 MAPK activation appeared mildly at early stage and reached a prominent degree at late time points (Fig. 1d; Supplementary Fig. S1d), whereas the activity of MKK3/6 and MKK4, upstream kinases known to phosphorylate p38 MAPK, was not significantly affected by GFAT1 deletion regardless of glucose availability, implying an alternative mechanism of p38 MAPK activation at this condition. In the meantime, treating A549 or H1299 cells with p38 MAPK inhibitor BIRB796 led to remarkable impairment of cell growth under glucose deprivation (Fig. 1e; Supplementary Fig. S1e). Of note, overexpression of MKK6E, the constitutively activated form of MMK6 that can lead to p38 activation, could significantly restore the cell growth upon GFAT1 deletion (Fig. 1f; Supplementary Fig. S1f), suggesting that the alternative way leading to p38 activation can also support cell survival under glucose deprivation. We wondered whether the regulation of p38 MAPK by GFAT1 was attributed to its metabolic activity. A549 cells with GFAT1 deletion were reconstituted with CRISPR-Cas9-resistant WT GFAT1 (WT rGFAT1) or the enzyme-inactive mutant GFAT1-H577A (rGFAT1-H577A) (Supplementary Fig. S1g, h). Consequently, GFAT1 deletion-suppressed cell growth was found notably rescued by WT rGFAT1 but not by rGFAT1-H577A expression under glucose deprivation (Fig. 1g; Supplementary Fig. S1i); similar effect was also verified in tumor growth analysis in nude mice (Fig. 1h), revealing the importance of metabolic activity of GFAT1 in cell growth. GFAT1 directly catalyzes production of glucosamine-6-phosphate (GlcN6P) with the conversion of glutamine into glutamate in HBP pathway (Fig. 1i). Enzymatic assay showed rGFAT1-H577A displayed an evidently decreased enzymatic activity (Supplementary Fig. S1j). Meanwhile, cellular overall O-GlcNAcylation level was conspicuously attenuated by GFAT1 deletion or rGFAT1-H577A expression in A549 cells, which was efficiently reversed with addition of a high dosage of exogenous glucosamine (GlcN) (Supplementary Fig. S1k). However, exogenous GlcN supplementation did not show sound rescue-effect on p38 MAPK phosphorylation or cell growth in GFAT1-deficient or rGFAT1-H577A-expressing cells under glucose deficiency (Fig. 1j; Supplementary Fig. S1l), suggesting that the enhancement of cellular O-GlcNAcylation alone is not sufficient for driving cell growth upon GFAT1 deletion. In contrast with this observation, addition of exogenous glutamate (Supplementary Fig. S1m) unexpectedly reversed the suppressed cell growth as well as p38 MAPK activation in rGFAT1-H577A-expressing cells rather than in GFAT1-deficient cells (Fig. 1j, k; Supplementary Fig. S1n). Of note, GFAT1 deletion exerted no sound effect on the intracellular glutamate and glutamine level, regardless of glucose availability (Supplementary Fig. S1o). These results indicate that the GFAT1 metabolic activity for glutamate production, instead of GlcN6P formation which fuels O-GlcNAcylation pathway, is indispensable for glucose deprivation-induced p38 MAPK activation; and the limited effect of exogenous glutamate in GFAT1-deficient cells implies that GFAT1 exerts additional regulatory effect that is independent of metabolic activity.

### TAB1 S438 phosphorylation is required for GFAT1–TAB1 interaction and p38 MAPK activation

**Fig. 2:**
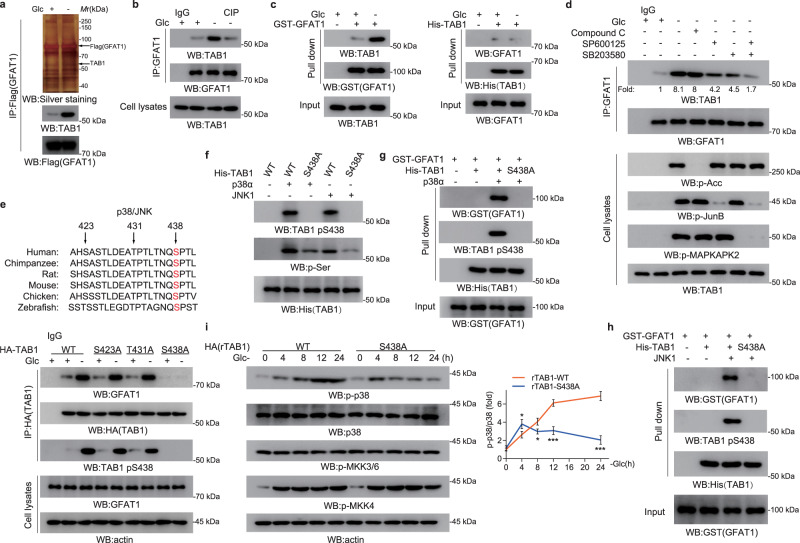
TAB1 interacts with GFAT1 in a Ser438 phosphorylation-dependent manner. **a** TAB1 could interact with GFAT1. A549 cells with stable expression of Flag-GFAT1 were cultured for 8 h under glucose deprivation. Immunoprecipitation and immunoblotting analyses were performed using the indicated antibodies. Silver staining analysis of the immunoprecipitates was performed. **b** TAB1–GFAT1 interaction was phosphorylation-dependent. A549 cells were cultured for 8 h under glucose deprivation. Immunoprecipitation and immunoblotting analyses were performed using the indicated antibodies. **c** TAB1 phosphorylation mediated TAB1–GFAT1 interaction. The indicated purified GST-GFAT1 or His-TAB1 protein was mixed with cell lysates from A549 cells cultured for 8 h under glucose deprivation. Pull-down and immunoblotting analyses were performed using the indicated antibodies. **d** JNK/p38 mediated TAB1–GFAT1 interaction. A549 cells were pretreated with Compound C (10 μM), SP600125 (20 μM), and SB203580 (10 μM) for 1 h before being cultured for 8 h under glucose deprivation. Immunoprecipitation and immunoblotting analyses were performed using the indicated antibodies. **e** Ser438 of TAB1 is evolutionarily conserved in the indicated species (upper panel). TAB1 Ser438 phosphorylation mediated TAB1–GFAT1 interaction. A549 cells with transient expression of WT TAB1, TAB1-S423A, TAB1-T431A or TAB1-S438A were cultured for 8 h under glucose deprivation. Immunoprecipitation and immunoblotting analyses were performed using the indicated antibodies (lower panel). **f** His-TAB1 Ser438 could be phosphorylated by p38α or JNK1. Purified His-TAB1 proteins were subjected to in vitro kinase assay. **g**, **h** In vitro binding of His-TAB1 and GST-GFAT1. Purified His-TAB1 WT and His-TAB1-S438A protein with phosphorylation by p38α (**g**) or JNK1 (**h**) was mixed with purified GST-GFAT1. Pull-down and immunoblotting analyses were performed using the indicated antibodies. **i** Expression of rTAB1-S438A inhibited p38 activation at late time points. A549 cells with depleted TAB1 and reconstituted expression of WT rTAB1 or rTAB1-S438A were cultured for indicated time under glucose deprivation. Immunoblotting analyses were performed using the indicated antibodies. In **i**, the values are presented as means ± SEM, *n* = 3; *P < 0.05, ***P < 0.001, Student’s *t*-test, two-sided.

To seek the concrete mechanism underlying the regulation of p38 MAPK activation by GFAT1, Flag-GFAT1 was stably expressed in A549 cells and the mass spectrometry analysis following Flag-GFAT1-immunoprecipitation was performed. As shown in Fig. 2a and Supplementary Fig. S2a, TAB1 was found to be specifically associated with GFAT1 under glucose deficiency. Consistent with the important role of TAB1 in p38 MAPK activation, glucose deprivation-induced p38 MAPK activation was tremendously inhibited by TAB1 depletion (Supplementary Fig. S2b). GFAT1–TAB1 interaction was further verified by co-immunoprecipitation at endogenous level in A549 and H1299 cells, in which the interaction was abolished by CIP treatment (Fig. 2b; Supplementary Fig. S2c), suggesting that protein phosphorylation is involved. The GST-pull down analysis showed that the purified recombinant GST-GFAT1 successfully bound to TAB1 from cellular extracts of A549 cells under glucose deficiency (Fig. 2c, left panel). Reciprocally, His-TAB1 showed no interaction with cellular GFAT1 regardless of glucose deprivation (Fig. 2c, right panel). These data suggest that the glucose deprivation-induced GFAT1–TAB1 interaction is more likely dependent on modification occurred in TAB1. To identify the key upstream signals required for the interaction, the inhibitors against AMPK (Compound C), p38 (SB203580), and JNK (SP600125) were utilized. The co-immunoprecipitation analysis showed that either SB203580 or SP600125 exhibited a moderately inhibitory effect on GFAT1–TAB1 interaction, whereas GFAT1–TAB1 complex formation was largely blocked when SB203580 and SP600125 are concomitantly used (Fig. 2d; Supplementary Fig. S2d), revealing that there is a synergic effect between p38 MAPK and JNK. Previous studies reported that TAB1 Ser423, Thr431 and Ser438 could be phosphorylated by p38 MAPK, among which Ser438 could also be phosphorylated by JNK^28^. We wondered TAB1 phosphorylation by p38 MAPK or JNK would be the prerequisite for GFAT1–TAB1 interaction. Intriguingly, mutagenesis analysis showed that the mutation of Ser438 (but not Ser423 or Thr431) into alanine greatly abolished GFAT1–TAB1 interaction under glucose deficiency (Fig. 2e). In line with this result, the protein kinase assay showed that either active p38α or JNK1 (Fig. 2f) could phosphorylate purified recombinant WT TAB1 but not TAB1-S438A, as verified by a specific TAB1 S438 phosphorylation antibody. Moreover, a GST-pull down analysis indicated that incubation of p38α or JNK1 with recombinant purified WT TAB1 but not TAB1-S438A successfully promoted GFAT1-binding ability of TAB1 (Fig. 2g, h). To further examine the effect of TAB1 S438 phosphorylation on p38 MAPK activation, A549 and H1299 cells with TAB1 depletion were reconstituted with shRNA resistant-WT TAB1 (WT rTAB1) or TAB1-S438A (rTAB1-S438A) (Supplementary Fig. S2e, f). As a result, p38 MAPK phosphorylation under glucose deficiency was evidently dropped at the late time points in cells with rTAB1-S438A expression (Fig. 2i; Supplementary Fig. S2g), but not under glutamine deficiency (Supplementary Fig. S2h). These results suggest that TAB1 S438 phosphorylation, which is required for GFAT1–TAB1 interaction, is crucial for sustained p38 MAPK activation under glucose deficiency.

### GFAT1 facilitates TTLL5-mediated TAB1 glutamylation required for p38 MAPK activation

**Fig. 3:**
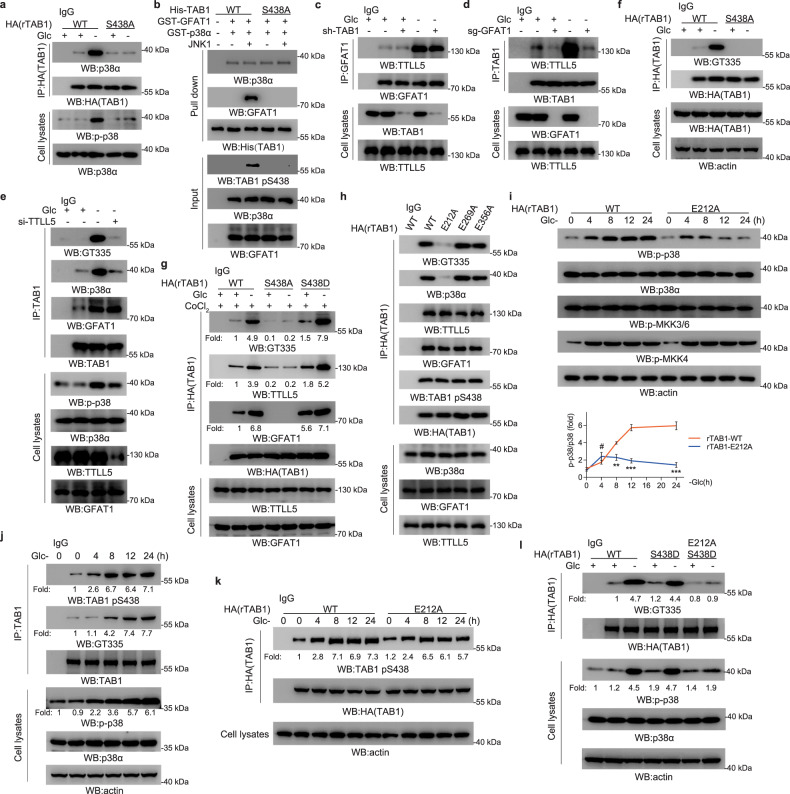
TAB1–GFAT1–TTLL5 complex promotes TAB1 glutamylation and p38 MAPK activation. **a** Expression of rTAB1-S438A inhibited TAB1–p38 interaction. A549 cells with depleted TAB1 and reconstituted expression of WT rTAB1 or rTAB1-S438A were cultured for 8 h under glucose deprivation. Immunoprecipitation and immunoblotting analyses were performed using the indicated antibodies. **b** TAB1 S438A mutation inhibited TAB1–p38 interaction in vitro. Purified His-TAB1 WT and His-TAB1-S438A protein with phosphorylation by JNK were mixed with purified GST-GFAT1 and GST-p38α. Pull-down and immunoblotting analyses were performed using the indicated antibodies. **c**, **d** GFAT1 mediated TAB1–GFAT1–TTLL5 complex formation. A549 cells with or without depleted TAB1 (**c**) or GFAT1 (**d**) were cultured for 8 h under glucose deprivation. Immunoprecipitation and immunoblotting analyses were performed using the indicated antibodies. **e** TTLL5 mediated TAB1 glutamylation. A549 cells transfected with or without TTLL5 siRNA were cultured for 8 h under glucose deprivation. Immunoprecipitation and immunoblotting analyses were performed using the indicated antibodies. **f** Expression of rTAB1-S438A inhibited TAB1 glutamylation. A549 cells with depleted TAB1 and reconstituted expression of WT rTAB1 or rTAB1-S438A were cultured for 8 h under glucose deprivation. Immunoprecipitation and immunoblotting analyses were performed using the indicated antibodies. **g** Expression of rTAB1-S438A inhibited TAB1 glutamylation in vitro. A549 cells with depleted TAB1 and reconstituted expression of WT rTAB1, rTAB1-S438A or rTAB1-S438D were cultured for 8 h under glucose deprivation with CoCl_2_ (10 μM). Immunoprecipitation, in vitro polyglutamylase assay and immunoblotting analyses were performed using the indicated antibodies. **h** TAB1 E212 was glutamylated. A549 cells with depleted TAB1 and reconstituted expression of WT rTAB1, rTAB1-E212A, rTAB1-E269A or rTAB1-E356A were cultured for 8 h under glucose deprivation. Immunoprecipitation and immunoblotting analyses were performed using the indicated antibodies. **i** Expression of rTAB1-E212A inhibited p38 activation at late time points. A549 cells with depleted TAB1 and reconstituted expression of WT rTAB1 or rTAB1-E212A were cultured for indicated time under glucose deprivation. Immunoblotting analyses were performed using the indicated antibodies. **j** Increased TAB1 S438 phosphorylation appeared earlier than increased TAB1 glutamylation. A549 cells were cultured for indicated time under glucose deprivation. Immunoprecipitation and immunoblotting analyses were performed using the indicated antibodies. **k** Expression of rTAB1-E212A inhibited TAB1 S438 phosphorylation at late time points. A549 cells with depleted TAB1 and reconstituted expression of WT rTAB1 or rTAB1-E212A were cultured for indicated time under glucose deprivation. Immunoprecipitation and immunoblotting analyses were performed using the indicated antibodies. **l** Glutamylation of rTAB1-S438D was blocked by E212A mutation. A549 cells with depleted TAB1 and reconstituted expression of WT rTAB1 or rTAB1-S438D or rTAB1-E212A/S438D were cultured for 8 h under glucose deprivation, immunoprecipitation and immunoblotting analyses were performed using the indicated antibodies. In **i**, the values are presented as means ± SEM, *n* = 3; ^#^*P* > 0.05, **P < 0.01, ***P < 0.001, Student’s *t*-test, two-sided.

It has been known that the complex formation between TAB1 and p38α MAPK can lead to p38α MAPK autophosphorylation and activation^24,29^. The co-immunoprecipitation analysis in A549 (Fig. 3a) and H1299 (Supplementary Fig. S3a) cells indicated that glucose deprivation apparently increased p38α MAPK binding to WT TAB1 but not TAB1-S438A, suggesting that TAB1 S438 phosphorylation-dependent GFAT1–TAB1 interaction is positively related to p38α MAPK recruitment to TAB1. However, the His-pull-down assay showed that JNK-mediated phosphorylation of TAB1 did not significantly affect TAB1–p38α interaction in vitro, although the binding of WT TAB1 instead of TAB1-S438A with GFAT1 was enhanced in the presence of JNK (Fig. 3b). To explain why GFAT1 is required here, we set forth to investigate the potential effect of GFAT1 on TAB1 protein status, which might be important for TAB1–p38α MAPK complex formation. Mass spectrometry analysis of Flag-GFAT1-precipitates also reveals TTLL5, which had an increased binding to GFAT1 under glucose deficiency (Supplementary Fig. S2a), as validated by the immnunoprecipitation (Supplementary Fig. S3b). TTLL5 belongs to a family of conserved proteins with a TTL homology domain that catalyzes ligation of glutamates to substrates^30,31^, modulating protein interaction of target protein^14,15^. Further analysis showed that glucose deprivation-induced endogenous GFAT1–TTLL5 and TAB1–TTLL5 association (Fig. 3c, d). GFAT1–TTLL5 interaction remained unaffected under TAB1 depletion (Fig. 3c), whereas the binding between TAB1 and TTLL5 was abrogated by GFAT1 deletion (Fig. 3d; Supplementary Fig. S3c). Consistently, glucose deprivation induced a comparable amount of GFAT1–TTLL5 complex in WT rTAB1- and rTAB1-S438A-expressing cells (Supplementary Fig. S3d). These data reveal that GFAT1 plays a central role in TAB1–GFAT1–TTLL5 complex formation.

Along with observations shown above, glucose deprivation induced a great increase of TAB1 glutamylation in a TTLL5-dependent manner, as detected by GT335, an antibody detects glutamylation (Fig. 3e). Meanwhile, glucose deprivation-induced TAB1 glutamylation was found to be blocked by GFAT1 deletion (Supplementary Fig. S3e), and TAB1-S438A but not WT TAB1 failed to undergo glutamylation (Fig. 3f). To investigate GFAT1-dependent TAB1 glutamylation mediated by TTLL5 in vitro, immunoprecipitates (anti-HA) from WT rTAB1-, rTAB1-S438A-, and rTAB1-S438D-expressing cells were subjected to in vitro glutamylase assay. As a result, rTAB1-S438D but not rTAB1-S438A showed enhanced binding to GFAT1 and TTLL5 under basal condition compared to WT rTAB1. In addition, elevated glutamylation level on WT rTAB1 and rTAB1-S438D under glucose deprivation further indicated that TAB1 glutamylation was dependent on TAB1/GFAT1/TTLL5 complex formation (Fig. 3g). In contrast, neither TAB1–p38α interaction nor TAB1 glutamylation was changed by glutamine deprivation (Supplementary Fig. S3f). Furthermore, glucose deprivation-induced TAB1 glutamylation and TAB1–p38α interaction remained unaffected with simultaneous glutamine deprivation for 8 h (Supplementary Fig. S3g).

To map the glutamylation site of TAB1, a mutagenesis analysis was performed and it showed that mutation of TAB1 E212 instead of the other 2 surface-located residues E269 or E356 (Supplementary Fig. S3h) into alanine dominantly decreased TAB1 glutamylation as well as the p38α MAPK-binding ability of TAB1, but without effect on TAB1 S438 phosphorylation (Fig. 3h). Given this, TAB1-depleted A549 (Supplementary Fig. S3i) and H1299 (Supplementary Fig. S3j) cells were reconstituted with shRNA resistant-TAB1-E212A (rTAB1-E212A) as well as WT rTAB1. In consistence with its effect on TAB1–p38α MAPK complex formation, rTAB1-E212A expression resulted in an attenuated tendency of p38 MAPK phosphorylation under glucose deficiency (Fig. 3i; Supplementary Fig. S3k), but not under glutamine deficiency (Supplementary Fig. S3l). The time course analysis showed that the notable increase of TAB1 S438 phosphorylation appeared earlier than that of TAB1 glutamylation under glucose starvation (Fig. 3j). Although TAB1-E212A expression could notably impair the p38 phosphorylation increase upon glucose deficiency (Fig. 3i), it only mildly attenuated TAB1 S438 phosphorylation at later time points (Fig. 3k). Therefore, it could be assumed that the limited effect of glutamylation abolishment on TAB1 S438 phosphorylation might due to the late occurrence of glutamylation and the complementary role of JNK for p38 in TAB1 S438 phosphorylation. Additionally, expression of TAB1 phosphor-mimic mutant TAB1-S438D that showed constitutive promotion of its binding to GFAT1 and its glutamylation (Fig. 3g), did not further increase p38 phosphorylation under glucose starvation (Fig. 3l). Of note, the inability of TAB1-S438D for p38 activation at basal level further revealed that TTLL5–TAB1 complex formation that only occurred under glucose starvation would also be importantly involved in the p38 activity regulation by GFAT1. Compared with that of TAB1-S438D, expression of the double mutant TAB1-E212A/S438D largely blocked cellular p38 activation under glucose starvation (Fig. 3l). These results suggest that GFAT1 facilitates TTLL5-mediated TAB1 glutamylation that subsequently promotes p38 MAPK phosphorylation.

### The metabolic activity of GFAT1 for glutamate production is required for TAB1 glutamylation

**Fig. 4:**
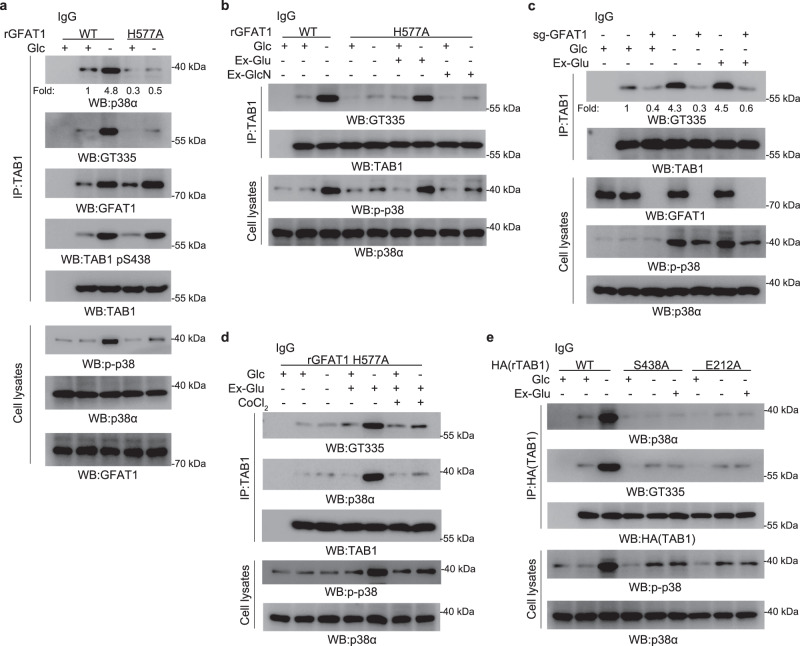
Glutamate derived from GFAT1 is required for TAB1 glutamylation. **a** Expression of rGFAT1-H577A inhibited TAB1 glutamylation and TAB1–p38 interaction. A549 cells with deleted GFAT1 and reconstituted expression of WT rGFAT1 or rGFAT1-H577A were cultured for 8 h under glucose deprivation. Immunoprecipitation and immunoblotting analyses were performed using the indicated antibodies. **b** Exogenous glutamate rescued TAB1 glutamylation in rGFAT1-H577A-expressing cells. A549 cells with deleted GFAT1 and reconstituted expression of WT rGFAT1 or rGFAT1-H577A were cultured for 8 h under glucose deprivation with exogenous glutamate (Ex-Glu) or glucosamine (Ex-GlcN). Immunoprecipitation and immunoblotting analyses were performed using the indicated antibodies. **c** Exogenous glutamate failed to rescue TAB1 glutamylation in GFAT1-deleted cells. A549 cells with or without deleted GFAT1 were cultured for 8 h under glucose deprivation with exogenous glutamate (Ex-Glu). Immunoprecipitation and immunoblotting analyses were performed using the indicated antibodies. **d** CoCl_2_ inhibited TAB1 glutamylation rescued by exogenous glutamate in rGFAT1-H577A-expressing cells. A549 cells with deleted GFAT1 and reconstituted expression of rGFAT1-H577A were cultured for 8 h under glucose deprivation with exogenous glutamate (Ex-Glu) and CoCl_2_ (10 μM). Immunoprecipitation and immunoblotting analyses were performed using the indicated antibodies. **e** Exogenous glutamate failed to rescue TAB1 glutamylation in rTAB1-S438A- or rTAB1-E212A-expressing cells. A549 cells with depleted TAB1 and reconstituted expression of WT rTAB1, rTAB1-S438A or rTAB1-E212A were cultured under glucose deprivation with exogenous glutamate (Ex-Glu). Immunoprecipitation and immunoblotting analyses were performed using the indicated antibodies.

Glutamylation modification is featured by its requirement of glutamate, which drives us to investigate whether GFAT1 activity for glutamate production contributes to TTLL5-mediated TAB1 glutamylation. As shown in Fig. 4a, either TAB1 glutamylation or TAB1–p38α MAPK interaction induced by glucose deprivation was evidently impaired by rGFAT1-H577A expression, although TAB1 S438 phosphorylation was unaffected. The co-immunoprecipitation analysis also indicated that GFAT1-H577A was able to bind to TTLL5 similar to WT GFAT1 under glucose deficiency (Supplementary Fig. S4a). In addition, treatment of high dosage of exogenous glutamate but not GlcN significantly rescued both TAB1 glutamylation and TAB1–p38α MAPK complex formation in rGFAT1-H577A-expressing cells (Fig. 4b; Supplementary Fig. S4b), which did not occurr in GFAT1-deleted cells (Fig. 4c). These results are in line with the repressive effect of GFAT1-H577A and the rescue-effect of exogenous glutamate on p38α MAPK phosphorylation (Fig. 1j). Meanwhile, the effect of exogenous glutamate on TAB1 glutamylation and TAB1–p38α interaction were found to be efficiently blocked by CoCl_2_ treatment used to augment cytosolic carboxypeptidases activity that can counteract TTLL5-mediated glutamylation^32^ (Fig. 4d). Together with the limited effect of exogenous glutamate observed in GFAT1-deleted cells shown here, these data further demonstrate that the physical effect of GFAT1 to facilitate TTLL5 activity against TAB1 is indispensable for TAB1 glutamylation. Consistent with this notion, addition of exogenous glutamate was unable to rescue TAB1-S438A- or TAB1-E212A-interupted TAB1–p38α interaction, TAB1 glutamylation and p38 activation (Fig. 4e). These results indicate that the metabolic activity of GFAT1 for glutamate production and GFAT1–TAB1 interaction are both required for TAB1 glutamylation under glucose deficiency.

### GFAT1/TAB1-dependent p38 activation promotes cancer cells survival under glucose deprivation via autophagy

**Fig. 5:**
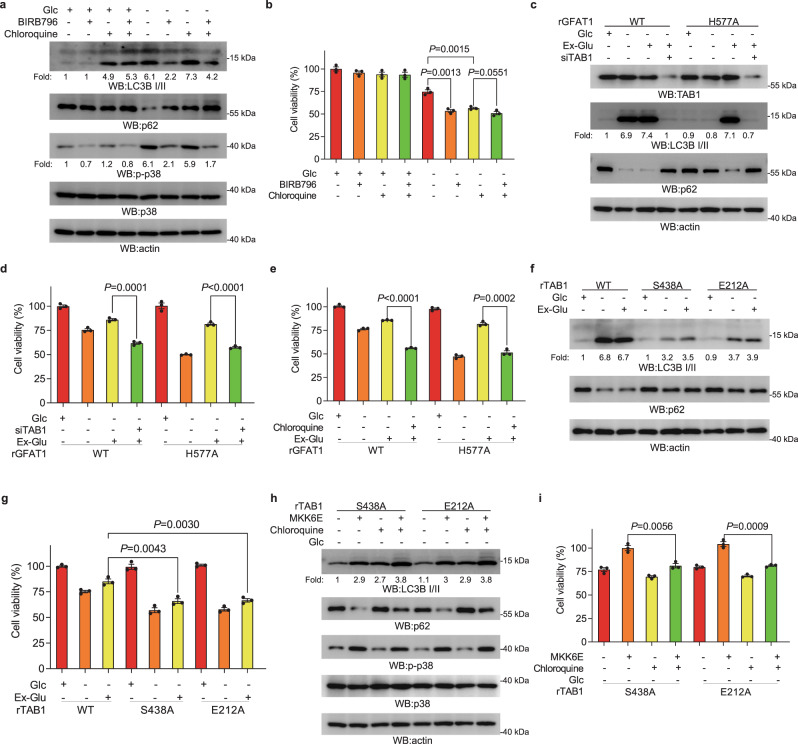
p38 MAPK activation is critical for autophagy and cell survival. **a** p38 inhibition suppressed autophagy. A549 cells were pretreated with BIRB796 (20 μM) and chloroquine (100 μM) for 1 h before being cultured for 8 h under glucose deprivation. Immunoblotting analyses were performed using the indicated antibodies. **b** p38 or autophagy inhibition suppressed cell survival. A549 cells were pretreated with BIRB796 (20 μM) and chloroquine (100 μM) for 1 h before being cultured under glucose deprivation. Cellular viability was examined by CCK8 assay at 36 h post glucose deprivation treatment. **c** Exogenous glutamate rescued autophagy in rGFAT1-H577A-expressing cells, but not when TAB1 was depleted. A549 cells with deleted GFAT1 and reconstituted expression of WT rGFAT1 or rGFAT1-H577A were transfected with TAB1 siRNA and cultured for 8 h under glucose deprivation with exogenous glutamate (Ex-Glu). Immunoblotting analyses were performed using the indicated antibodies. **d** Exogenous glutamate rescued cell survival in rGFAT1-H577A-expressing cells, but not when TAB1 was depleted. A549 cells with deleted GFAT1 and reconstituted expression of WT rGFAT1 or rGFAT1-H577A were transfected with TAB1 siRNA and cultured for 8 h under glucose deprivation with exogenous glutamate (Ex-Glu). Cellular viability was examined by CCK8 assay at 36 h post glucose deprivation treatment. **e** Autophagy inhibition suppressed cell survival rescued by exogenous glutamate in rGFAT1-H577A-expressing cells. A549 cells with deleted GFAT1 and reconstituted expression of WT rGFAT1 or rGFAT1-H577A were pretreated with chloroquine (100 μM) for 1 h before being cultured under glucose deprivation with exogenous glutamate (Ex-Glu). Cellular viability was examined by CCK8 assay at 36 h post glucose deprivation treatment. **f** Exogenous glutamate failed to rescue autophagy in rTAB1-S438A- or rTAB1-E212A-expressing cells. A549 cells with depleted TAB1 and reconstituted expression of WT rTAB1, rTAB1-S438A or rTAB1-E212A were cultured for 8 h under glucose deprivation with exogenous glutamate (Ex-Glu). Immunoblotting analyses were performed using the indicated antibodies. **g** Exogenous glutamate failed to rescue cell survival in rTAB1-S438A- or rTAB1-E212A-expressing cells. A549 cells with depleted TAB1 and reconstituted expression of WT rTAB1, rTAB1-S438A or rTAB1-E212A were cultured under glucose deprivation with exogenous glutamate (Ex-Glu). Cellular viability was examined by CCK8 assay at 36 h post glucose deprivation treatment. **h** Chloroquine blocked autophagy rescued by expression of MKK6E in rTAB1-S438A- or rTAB1-E212A-expressing cells. A549 cells with depleted TAB1 and reconstituted expression of rTAB1-S438A or rTAB1-E212A were transfected with MKK6E and pretreated with chloroquine (100 μM) for 1 h before being cultured for 8 h under glucose deprivation. Immunoblotting analyses were performed using the indicated antibodies. **i** Chloroquine blocked cell survival rescued by expression of MKK6E in rTAB1-S438A- or rTAB1-E212A-expressing cells. A549 cells with depleted TAB1 and reconstituted expression of rTAB1-S438A or rTAB1-E212A were transfected with MKK6E and pretreated with chloroquine (100 μM) for 1 h before being cultured under glucose deprivation. Cellular viability was examined by CCK8 assay at 36 h post glucose deprivation treatment. In **b**, **d**, **e**, **g**, and **i**, the values are presented as means ± SEM, *n* = 3; *P* values (Student’s *t*-test, two-sided) with control or the indicated groups are presented.

Glucose deprivation can stimulate autophagy by which cells may temporarily recycle cytoplasmic material to overcome nutritional stresses. It has been known that p38 activation is involved in stress-induced autophagy^33–35^. We further investigated whether autophagy is regulated as the downstream response of the GFAT1-TAB1-p38 axis to affect cell survival. Under glucose deprivation, treatment of either BIRB796 or chloroquine, an inhibitor used to restrict fusion of autophagosome and lysosome, notably suppressed autophagy as indicated by the impaired LC3II accumulation or p62 degradation (Fig. 5a), which was accompanied by the impaired cell growth (Fig. 5b; Supplementary Fig. S5a). In comparison, simultaneous treatment of chloroquine and BIR796 did not show the significantly addictive effect on autophagy and cell growth (Fig. 5b; Supplementary Fig. S5a), revealing that under glucose starvation p38 activation promotes lung adenocarcinoma cell survival and this is closely related to autophagy. In addition, glucose deprivation-induced LC3II accumulation was apparently attenuated by expression of rGFAT1-H577A compared to WT rGFAT1, and the attenuated LC3II was rescued to certain degree by chloroquine treatment (Supplementary Fig. S5b). Meanwhile, attenuated LC3II accumulation under rGFAT1-H577A expression was found to be evidently restored by addition of exogenous glutamate instead of GlcN (Supplementary Fig. S5c). Of note, TAB1 depletion attenuated glucose deprivation-induced autophagy in cells with intact function of GFAT1 (Supplementary Fig. S5d), and blocked the rescue-effect of exogenous glutamate on autophagy in rGFAT1-H577A-expressing cells (Fig. 5c; Supplementary Fig. S5e). These results suggest that GFAT1-catalyzed glutamate generation is required for autophagy initiation. Functionally, expression of rGFAT1-H577A resulted in decreased cell viability compared with WT group under glucose deficiency, which was soundly rescued by exogenous glutamate in a TAB1-dependent manner (Fig. 5d; Supplementary Fig. S5f). Meanwhile, the enhanced viability of rGFAT1-H577A-expressing cells by exogenous glutamate was also impeded by chloroquine treatment, further indicating that glutamate supplementation supports cell viability through its positive effect on autophagy (Fig. 5e; Supplementary Fig. S5g). In addition, we found that expression of rTAB1-S438A, as well as rTAB1-E212A dramatically suppressed autophagy and cell viability compared with the WT counterpart under glucose deficiency, and this effect could not be significantly rescued by addition of exogenous glutamate (Fig. 5f, g; Supplementary Fig. S5h, i). We wonder the distinct effect of exogenous glutamate on autophagy and cell growth among rGFAT1-H577A-expressing and rTAB1-S438A- or rTAB1-E212A-expressing cells is attributed to its relevant effect on p38 activation. In line with this assumption, overexpression of MKK6E, which constitutively activates p38, effectively promoted autophagy and cell survival in rTAB1-S438A- or rTAB1-E212A-expressing cells under glucose deficiency (Fig. 5h, i), while the enhanced cell survival by MKK6E was inhibited by chloroquine treatment. These results indicate that GFAT1/TAB1-dependent p38 activation promotes autophagy and cell growth under nutrient stress. In addition, it was found that under glucose deprivation the cell proliferation was attenuated by expression of TAB1-S438A or TAB1-E212A (Supplementary Fig. S5j), which was accompanied with the apoptotic events as indicated by the increased cleaved-caspase3 level (Supplementary Fig. S5k); these effects were found to be counteracted by MKK6E overexpression. These data support that the positive effect of p38 activation (mediated by GFAT1–TAB1 complex under glucose starvation) on cell survival might be attributed to its multiple physiological roles that either in cell apoptosis or in autophagy.

### TAB1 S438 phosphorylation promotes tumorigenesis and is associated with poor prognosis in lung adenocarcinoma

**Fig. 6:**
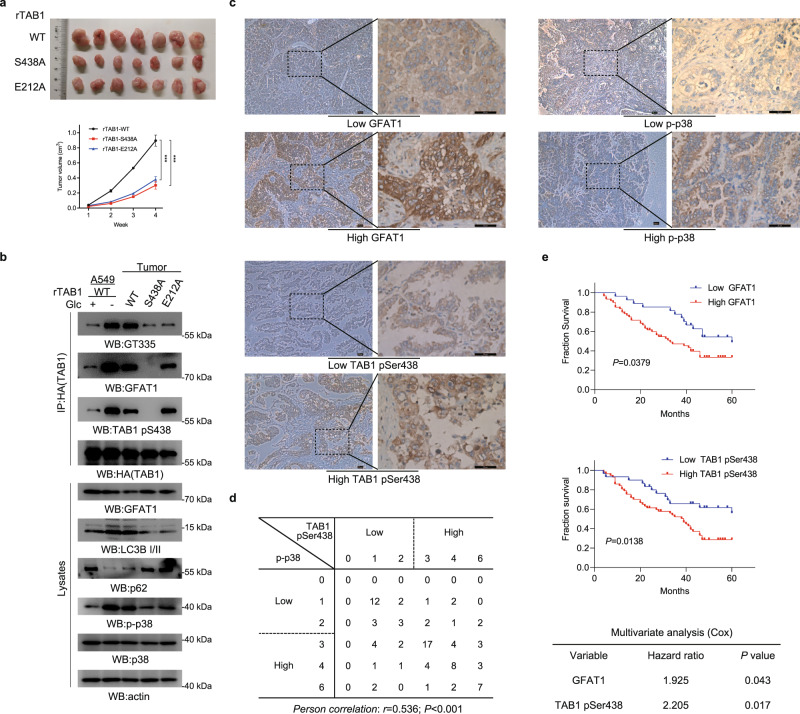
GFAT1-TAB1-TTLL5 axis promotes tumorigenesis and is related to poor prognosis. **a** Expression of rTAB1-S438A or rTAB1-E212A suppressed tumorigenesis. A total of 5 × 10^6^ A549 cells with TAB1 depletion and reconstituted expression of the WT rTAB1, rTAB1-S438A or rTAB1-E212A were subcutaneously injected into athymic nude mice. Representative tumour xenografts are shown (upper panel). Tumor volumes were measured by using length “a” and width “b” and calculated using the following equation: V = ab^2^/2. **b** Expression of rTAB1-S438A or rTAB1-E212A suppressed TAB1-p38-autophagy axis. Immunoprecipitated HA-rTAB1 from lysates of A549 cells that were cultured for 8 h under glucose deprivation and 7 pooled tumor tissues (as indicated in **a**) was subjected to immunoblotting analyses using the indicated antibodies. **c** Immunohistochemical staining was performed on 87 human lung adenocarcinoma specimens using the indicated antibodies. Representative images are shown. Scale bars, 50 µm. **d** Semi-quantitative scoring and correlation analysis indicating the correlation between p-p38 and TAB1 pSer438 (Person correlation test; r = 0.536, *P* < 0.001). **e** The survival durations for 87 patients with low (0–2 staining scores, blue curve) versus high (3–6 staining scores, red curve) GFAT1 (low, *n* = 27 patients; high, *n* = 60 patients) and with low (0–2 staining scores, blue curve) versus high (3–6 staining scores, red curve) TAB1 Ser438 phosphorylation (low, *n* = 30 patients; high, *n* = 57 patients) were compared. The Kaplan–Meier method and log-rank tests indicate the significance level of the association of GFAT1 (*P* = 0.0379) with patient survival and the significance level of the association of TAB1 Ser438 phosphorylation (*P* = 0.0138) with patient survival. The table shows the Cox multivariate analysis after adjustment for patient sex and age, indicating the significance level of the association of GFAT1 (*P* = 0.043, HR = 1.925) with patient survival and the significance level of the association of TAB1 Ser438 phosphorylation (*P* = 0.017, HR = 2.205) with patient survival. In **a**, the values are presented as means ± SEM, *n* = 7; ***P < 0.001, Student’s *t*-test, two-sided.

To validate the significance of the regulatory effect of GFAT1 on TAB1-dependent p38 activation in tumorigeneisis, athymic nude mice were injected with A549 cells expressing WT rTAB1, rTAB1-S438A, or rTAB1-E212A respectively. We found that disruption of either JNK/p38-mediated phosphorylation or TAB1 glutamylation led to a suppressed tumor growth (Fig. 6a). The following immunoblotting analysis indicated that phosphorylation levels of TAB1, p-p38 level and autophagy flux in tumor tissues from WT group were notably enhanced in comparison with their basal levels shown in A549 cells cultured in vitro (Fig. 6b). The p38 activation was apparently reduced in tumor tissues derived from cells expressing rTAB1-S438A, or rTAB1-E212A. These results suggest that both TAB1 phosphorylation and glutamylation are required for p38 activation and tumor growth in vivo.

The positive correlation of p38 MAPK activation with lung adenocarcinoma malignancy has been reported^20^. Our mechanistic study shows that GFAT1 along with TAB1 S438 phosphorylation contributes to p38 MAPK activation, thus we performed IHC analyses in serial sections of 87 human lung adenocarcinoma specimens to examine the clinical relevance of GFAT1, p-p38 level and TAB1 S438 phosphorylation levels (Fig. 6c). The specificity of TAB1 S438 phosphorylation antibody was confirmed by IHC analyses with specific blocking peptide (Supplementary Fig. S6a). Semi-quantitative analyses showed the positive correlation between p-p38 level and TAB1 S438 phosphorylation level (Fig. 6d). Further clinical analysis showed the Pearson correlation coefficient between GFAT1 and TAB1 S438 phosphorylation or p-p38 only reached a middle value (Supplementary Fig. S6b, c), which may be due to the reason that the expressions of GFAT1, TAB1 pS438 or p-p38 can be influenced independently by additional factors besides glucose availability. The survival duration of patients was evaluated with respect to the levels of GFAT1 or TAB1 S438 phosphorylation. Patients whose tumors displayed low GFAT1 expression (27 cases) had a median survival duration of 60 months; those whose tumors displayed high levels of GFAT1 (60 cases) had a significantly lower median survival duration of 33 months (Fig. 6e). Meanwhile, patients whose tumors had low TAB1 S438 phosphorylation level (30 cases) had a significantly higher median survival duration that was not reached; those whose tumors had high levels of TAB1 S438 phosphorylation (57 cases) had a median survival duration of 39 months (Fig. 6e). The cox-multivariate analysis with patient sex and age adjustment indicates the levels of GFAT1 and TAB1 S438 phosphorylation were significantly associated with patient survival (Fig. 6e). These data reveal that high GFAT1 or TAB1 S438 phosphorylation predicts a poor prognosis for human lung adenocarcinoma.

## Discussion

**Fig. 7:**
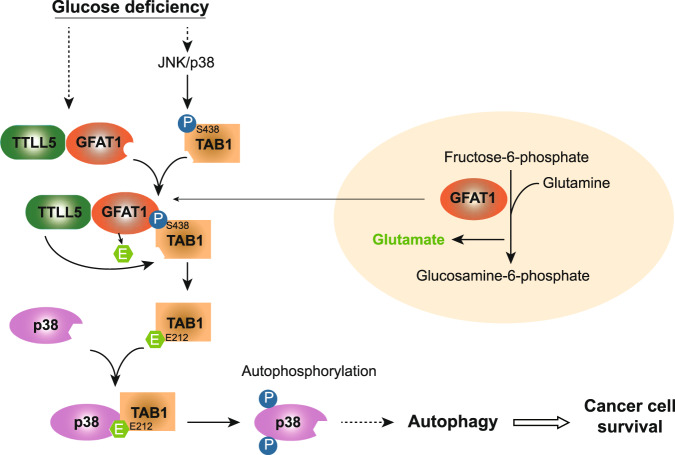
A mechanism underlying GFAT1-drived cancer cell survival under glucose starvation. A schematic model showing the molecular basis of GFAT1-TAB1-p38 signaling in autophagy-induced cancer cell survival. Glucose deficiency promotes TAB1 S438 phosphorylation by JNK/p38 MAPK and subsequent TTLL5–GFAT1–TAB1 complex formation. GFAT1-derived glutamate facilitates TAB1 E212 glutamylation through TTLL5, resulting in enhanced TAB1-p38 MAPK interaction and p38 MAPK autophosphorylation. GFAT1-TAB1-p38 MAPK axis sustains cancer cell survival under glucose deprivation via autophagy.

In addition to the canonical pathway that involves the upstream kinases MKKs^23^, p38 activation can circumstantially occurs in MKKs-independent manner, in which TAB1, identified as a TAK1 binding partner, is able to bind to p38 MAPK and promotes its autophosphorylation^24,25,35^. The indispensable role of TAB1 in p38 autophosphorylation is observed in distinct tissues under nutrient stress^26,35^; in this regard, there remains debate about whether AMPK, a master energy senor, is involved in TAB1–p38 MAPK interaction, and it seems that AMPK is engaged into TAB1-mediated p38 MAPK activation in context-dependent manner^26,36,37^. Physiologically, the biological effect of p38 activation is complicated, which to some degree would be attributed to the complicacy of its regulatory mechanism. In the present study, we show that glucose deprivation triggers TAB1 S438 phosphorylation, which enables TAB1 to bind to GFAT1 and results in the TTLL5–GFAT1–TAB1 complex formation. Importantly, GFAT1-catalized production of glutamate feeds into TTLL5-mediated TAB1 glutamylation, which facilitates TAB1 recruitment to p38 MAPK and thereby activates p38 MAPK (Fig. 7). Thus, our finding uncovers an unidentified regulatory mechanism of TAB1-mediated p38 activation that involves the metabolic enzyme GFAT1. In detail, time-course analysis indicates that it is more likely that GFAT1 contributes to the persistence of p38 MAPK phosphorylation under glucose deficiency; this is consistent with the observation that besides JNK, p38 MAPK is also able to phosphorylate TAB1 Ser438, which means the initial activation of p38 MAPK would be required for TAB1 S438 phosphorylation induction and the subsequent GFAT1 engagement, and GFAT1-TAB1-p38 MAPK axis could act as a positive feedback loop for p38 MAPK autophosphorylation.

As the rate-limiting enzyme of HBP, GFAT1 exerts catalytic activity for GlcN6P and glutamate formation using fructose-6-phosphate and glutamine as substrates. The local metabolic effect of GFAT1 on TAB1 glutamylation through glutamate production makes the precise regulation of TAB1. The efficient allocation of GFAT1 activity into this nutrient stress-responsive event would be essential since under glucose deficiency the carbon source for fructose-6-phosphate synthesis accordingly becomes limited. On the other hand, GFAT1 expression level is found to be upregulated upon glucose starvation, it implies the requirement of high amount of GFAT1 for energy stress-relevant cellular activities that might include the regulatory effect of GFAT1 on TAB1. In addition, the basal level of GFAT1 is shown to be largely enhanced in tumors compared to normal tissues. It also can be inferred that the GFAT1-TAB1-p38 MAPK axis is augmented in cancer cells as supported by the data of our functional studies confers the strong resistance of cancer cells to growth inhibition induced by glucose deficiency. The time course analysis indicated glucose deprivation and glutamine deprivation resulted in distinct effects on GFAT1 expression level (Supplementary Fig. S1b, c). It is well-known that both glucose and glutamine are vital carbon sources to support unlimited growth of cancer cells, and each of them is able to trigger signals linking to cell homeostasis including the regulation of metabolic enzymes^2^. For instance, GFAT1 expression is upregulated by X-Box Binding Protein 1, a transcription factor functions during ER stress^38^. It could be inferred that glucose deprivation-induced unfolded protein response might contribute to increased expression of GFAT1 observed in our study. On the other hand, it has been found that the downregulation of GFAT1 expression by glutamine deprivation was related to the restrained TCA cycle at this condition^8^. Our results suggest that glutamine utilized by GFAT1 through its protein complex-proximal activity is critical for sustained p38 activation upon glucose deficiency. However, the concrete mechanism underlying the influence of glucose or glutamine abundance on GFAT1 expression is worth of further investigation.

Previous studies reveal that p38 MAPK signaling can exert either anti-tumorigenic or pro-tumorigenic effects^39^, and the relationship between p38 MAPK and cancer development should be estimated with the consideration of the tissue type, genetic background, and stage of tumors. With respect to lung adenocarcinoma, the high p38 MAPK activity is reported to be specifically detected in tumor tissues compared with the normal counterparts^20^. Here, we found that GFAT1 facilitates TAB1-coupled p38 MAPK activation and contributes to lung adenocarcinoma cell survival under glucose deficiency through its positive effect on autophagy occurrence, pointing out GFAT1 could be a potential therapeutic target against lung adenocarcinoma. P38 MAPK may affect autophagy via multiple mechanism upon stress stimulus^34,40,41^, among which the effector related to ER stress-induced autophagy is found to be regulated by p38 MAPK specifically at lysosomes. It could be inferred that the concrete biological outcome of autophagy-related p38 MAPK activation would be also determined by specific organelle localization of the upstream and downstream effectors. Thus, further investigation of the subcellular distribution of GFAT1-TAB1-p38 MAPK signal and identification of its compartmental downstream effectors will be beneficial for better understanding the impact of GFAT1-mediated p38 MAPK activation in tumorigenesis.

## Materials and Methods

### Cell culture

Human NSCLC cell lines A549 and H1299 were obtained from Shanghai Cell Bank (Shanghai Biological Sciences, Chinese Academy of Sciences, Shanghai, China) and were originally obtained from the ATCC, authenticated by short tandem repeat profiling within 6 months. Cells were maintained in RPMI1640 supplemented with 10% FBS.

### DNA constructs and mutagenesis

The DNA constructs and mutagenesis were performed as previously described^42^. The sequence of Flag-tag of GFAT1 in the plenti-IRES vector is located at the C-terminus of GFAT1. lentiCRISPRv2 human GFAT1 sgRNA was generated with the oligonucleotides 5′-GCGCCGACACGACTCCCTCGGGG-3′ and 5′-TGGAATAGCTCATACCCGTTGGG-3′. pGIPZ human TAB1 shRNA was generated with the oligonucleotides 5′-GGGGATTACAAGGTTAAAT-3′ and 5′-ACGAGGACATGACCCTGCT-3′. Human TTLL5 siRNAs were generated with the oligonucleotides 5′-CGAGGUGGAUUUAUUCGCAUAUU-3′; 5′-UAUGCGAAUAAAUCCACCUCGUU-3′. Human JNK siRNAs were generated with the oligonucleotides 5′-UUCUAGAAACCCAUAAGGCCA-3′; 5′-AAGCCCAGUAAUAUAGUAGUA-3′.

### Antibody

Antibodies that recognize GFAT1 (ab125069, 1:1000), p38α (ab59461, 1:1000), TTLL5 (ab187697, 1:1000), TAB1 (ab76412, 1:1000) and β-actin (ab8227, 1:1000) were purchased from Abcam. Antibodies that recognize p62 (sc-28359, 1:1000), O-GlcNAc (sc-59623, 1:1000) LC3B (sc-271625, 1:1000) and p-p38 (sc-7973, 1:1000) were obtained from Santa Cruz. Antibody against Flag (F1804, 1:1000) was purchased from Sigma. Antibodies against p-MAPKAPK-2 (#3044, 1:1000), p-JunB (#8053, 1:1000), p-Acc (#11818, 1:1000), AMPK (#2603, 1:1000), JNK (#9252, 1:1000), p38α (#9218, 1:1000), p-AMPK (#50081, 1:1000), p-JNK (#4668, 1:1000), p-MKK3/6 (#9231, 1:1000), LC3B (#3868, 1:1000), p-p38 (#4511, 1:1000), His (#9991, 1:5000), GST (#5475, 1:5000) and O-GlcNAc (#9875, 1:1000) were purchased from Cell Signaling Technology. Antibodies that recognize TAB1 (D122651, 1:1000), HA (D191044, 1:1000) were purchased from Sangon Biotech. Antibody GT335 was purchased from Adipogen. Antibodies that recognize p38α (#48644, 1:1000) and β-actin (#49294, 1:1000) were purchased from Signalway Antibody. Phospho Ser438 TAB1 antibody (AF8324, 1:1000) was purchased from Affinity. GFAT1 antibody (14132-1-AP, 1:1000) and Caspase 3 antibody (19677-1-AP) were purchased from Proteintech.

### Cell proliferation assay

Cell proliferation assay was carried out as described previously^42^. Briefly, after treatment, cells were incubated in CCK8 (10%v/v, 40203ES60, YEASEN) that was diluted in normal culture medium at 37 °C for 0.5–4 h until the visual color conversion occurred. The samples were then analyzed by measuring the absorbance of 450 nm using FLx800 Fluorescence Microplate Reader (Biotek).

### Transfection

A549 and H1299 Cells were transfected with various plasmids using lipofectamine 3000 (Invitrogen) according to the manufacture’s instructions.

### Immunoprecipitaion and immunoblotting

Immunoprecipitaion and immunoblotting were performed as described previously^42^. Briefly, cells were lysed in lysis buffer (50 mM Tris-HCl pH 7.4, 150 mM NaCl, 1 mM EDTA, 1% NP-40, 1% sodium deoxycholate, and 0.1% SDS and cocktails of proteinase and phosphatase inhibitors), followed by immunoprecipitation and immunoblotting. For immunoprecipitation, cell lysates were centrifuged at 12,000 rpm for 10 min and the supernatants were pre-cleared with protein G beads, and then incubated with indicated antibodies and protein G beads overnight at 4 °C. The proteins were eluted with peptide after extensive washing, then boiled in SDS-PAGE sample buffer. For immunoblotting, cell lysates were separated on SDS-PAGE gels and transferred to PVDF membranes. Membranes were blocked with 5% milk or BSA, then incubated with indicated antibodies and exposed to horseradish peroxidase (HRP)-conjugated secondary antibody.

### Immunohistochemistry

Immunohistochemistry was performed on paraffin-embedded sections of human lung carcinoma samples as described previously^42^. Briefly, after deparaffinized and rehydrated, antigen retrieval was performed by boiling tissue sections in 10 mM citrate buffer (pH 6.0) in a microwave oven for 5 min. The activity of endogenous peroxidase was blocked with 3% hydrogen peroxide in methanol for 10 min at room temperature. After washing, non-specific binding sites were blocked by incubating the slides with 10% FBS/PBS for 30 min at room temperature. Sections were subsequently incubated with anti-GFAT1 (Proteintech, 14132-1-AP, 1:100) and anti-TAB1 pS438 (Affinity, AF8324, 1:50) at 4 °C overnight. After incubation with the primary antibodies, the sections were washed and incubated with secondary antibodies and DAB staining reagent with GTVisionTM Detection System/Mo&Rb Kit according to manufacturer’s instructions. After counterstain with hematoxylin and dehydration, the sections were mounted and imaged using the Leica microscope.

Immunoreactivity was semi-quantitatively evaluated according to intensity and area: the staining intensity of lung adenocarcinoma cells was recorded as “no staining” (0), “weak to moderate staining” (1) or “strong staining” (2). The area of stained cancer cells was recorded as < 33% (1), 33%–66% (2) or > 66% (3) of all cancer cells. These numbers were then multiplied, resulting in a score of 0–6.

Tissue microarray of human lung adenocarcinoma and paired adjacent normal tissues (HLugA180Su04) were purchased from Shanghai Outdo Biotech Company (Shanghai, China). The study was approved by the Ethics Committee of Shanghai Outdo Biotech Company.

### Glutamate measurement

Concentration of glutamate was measured using commercial ELISA kit, which was obtained from Solarbio (BC1585). The experiments were carried out according to the operating manual.

### Cell proliferation assay

EdU labeling and staining was performed using an EdU kit (BeyoClick™ EdU Cell Proliferation Kit with Alexa Fluor 488, Beyotime, China). The experiments were carried out according to the operating manual.

### Recombinant protein purification

His-TAB1 and GST-GFAT1 were expressed in bacteria and purified, as described previously^42^. Briefly, corresponding expression constructs were transformed into BL21 (DE3) bacteria. The bacteria were cultured at 37 °C to an OD600 of 0.6–1 before inducing with IPTG (0.1 mM) for 6 h. Cultures were centrifuged and pellets were lysed by sonication. Cleared lysates were bound to HisSep Ni-NTA Agarose Resin or GST-Sefinose (TM) Resin, respectively. Eluates were concentrated by using Ultrafree-15 centrifugal filters (Millipore, Bedford, MA).

### Enzyme activity assay

The protocol of enzyme activity assay was modified from previous studies^43^. Purified WT or mutant GFAT1was added to enzyme assay buffer (0.8 mM fructose-6-phosphate; 6.0 mM, glutamine; 50 mM KCl; 0.1 mM KH_2_PO_4_; pH 7.8) before incubated at 37 °C for 1 h. The concentration of glutamate was measured.

### In vitro kinase assay

Purified recombinant WT and mutant His-TAB1 from bacteria were incubated with p38α/JNK1 in kinase assay buffer supplemented with 75 mM β-glycerophosphate (pH 7.3), 3.75 mM EGTA, 30 mM MgCl_2_, 4.5 mM DTT, 0.15 mM Na_3_VO_4_, 0.3 mM ATP at 30 °C for 1 h. After the reaction, p38α/JNK1 was removed by extensive washing with RIPA buffer and kinase assay buffer. The His-TAB1 bound beads after JNK1 treatment in the absence of hot ATP were further incubated with GST-GFAT1 or p38α for protein interaction assay as indicated.

### In vitro glutamylase assay

Cells stably expressing HA-rTAB1-WT/S438A/S438D were treated with CoCl_2_ (10 μM) before immunoprecipitaion using anti-HA antibody. The precipitates were added to the reaction buffer (50 mM Tris-HCl, pH 7.0, 2 mM ATP, 10 mM MgCl_2_, 2.5 mM dithiothreitol, 20 μM Glutamate) and incubated at 30 °C for 1 h. The reaction was stopped by boiling in SDS loading buffer for 5 min.

### 3D structure model

The data of crystal structure of TAB1 were obtained from PROTEIN DATA BANK (PDB) and processed by PyMOL.

### Mice

Five-week-old male nu/nu mice (7 per group) were injected with 5 × 10^6^ A549 cells with indicated treatments in 150 mL PBS. All animal experiments were approved by the animal care and use committee of Guangzhou Medical University.

### Statistics and reproducibility

Unpaired two-tailed Student’s *t*-test was used for statistical analysis. All experiments were performed at least three times unless otherwise indicated. Analyses were performed with the SPSS 22.0 and Graphpad Prism 8.0 statistical analysis software. Statistical significance was defined as *P* < 0.05.

## Supplementary information

Supplementary figures

## Data Availability

The data that support the findings of the study are available from the corresponding author upon reasonable request. Informed consent for publication was obtained from all the authors.
