# Supplementary material for: GFAT1-linked TAB1 glutamylation sustains p38 MAPK activation and promotes lung cancer cell survival under glucose starvation
Source: Cell Discov. 2022 Aug 9;8:77. doi: 10.1038/s41421-022-00423-0 (PMC9363421; doi:10.1038/s41421-022-00423-0)
Supplement: Supplementary file 1 — Supplementary figures [file 41421_2022_423_MOESM1_ESM.pdf]

S1

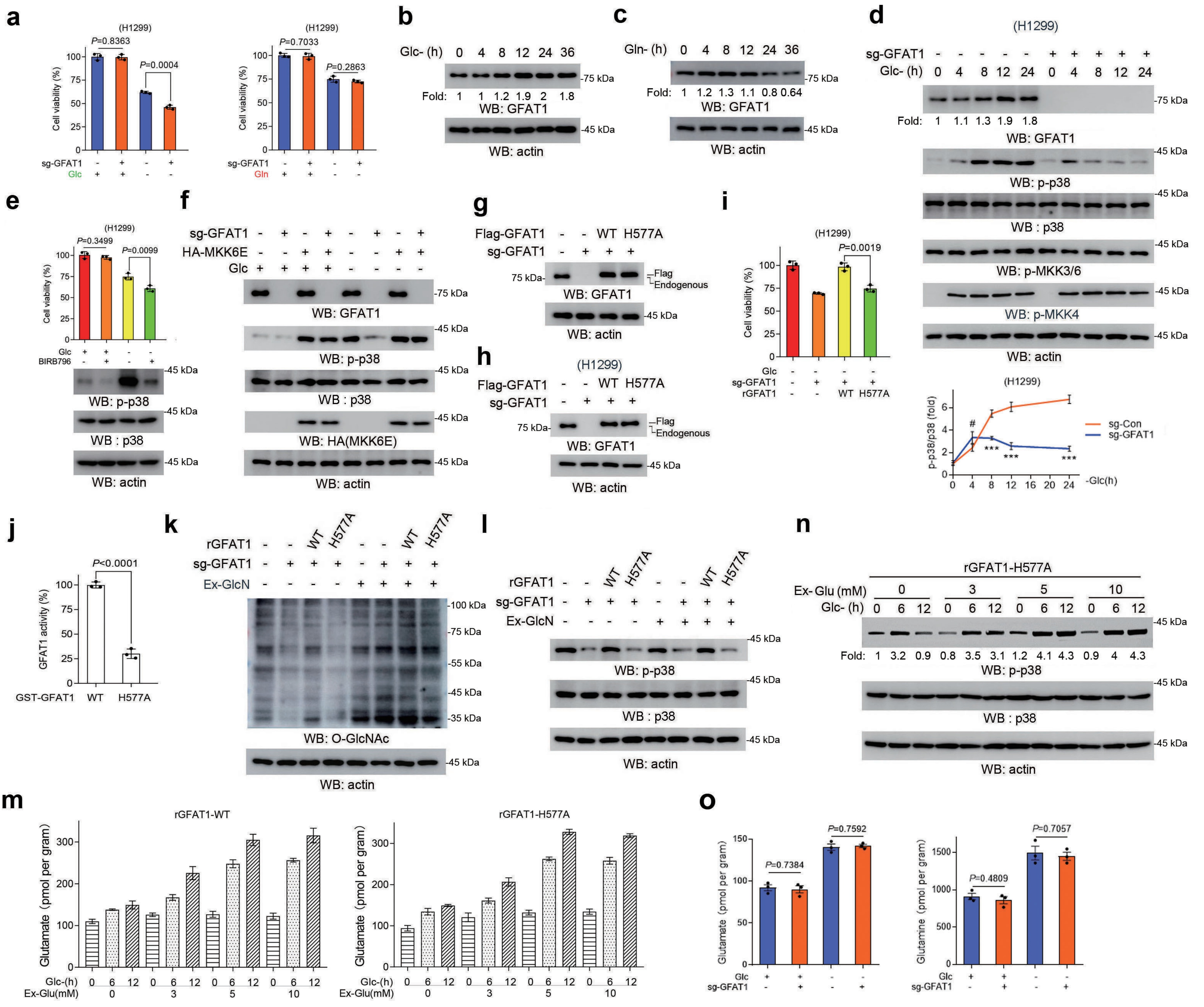
a
(H1299)
P=0.8363
P=0.0004
Cell viability (%)
sg-GFAT1
Glc
(H1299)
P=0.7033
P=0.2863
Cell viability (%)
sg-GFAT1
Gln
b
Glc- (h) 0 4 8 12 24 36
-75 kDa
Fold: 1 1 1.2 1.9 2 1.8
WB: GFAT1
-45 kDa
WB: actin
c
Gln- (h) 0 4 8 12 24 36
-75 kDa
Fold: 1 1.2 1.3 1.1 0.8 0.64
WB: GFAT1
-45 kDa
WB: actin
d
(H1299)
sg-GFAT1
Glc- (h) 0 4 8 12 24 0 4 8 12 24
-75 kDa
Fold: 1 1.1 1.3 1.9 1.8
WB: GFAT1
WB: p-p38
WB : p38
WB: p-MKK3/6
WB: p-MKK4
WB: actin
(H1299)
p-p38/p38 (fold)
sg-Con
sg-GFAT1
-Glc(h)
e
(H1299)
P=0.3499
P=0.0099
Cell viability (%)
Glc
BIRB796
WB: p-p38
WB : p38
WB: actin
f
sg-GFAT1
HA-MKK6E
Glc
WB: GFAT1
WB: p-p38
WB : p38
WB: HA(MKK6E)
WB: actin
g
Flag-GFAT1 - - WT H577A
sg-GFAT1
75 kDa-
Flag
Endogenous
WB: GFAT1
WB: actin
h
(H1299)
Flag-GFAT1 - - WT H577A
sg-GFAT1
75 kDa-
Flag
Endogenous
WB: GFAT1
WB: actin
i
(H1299)
P=0.0019
Cell viability (%)
Glc
sg-GFAT1
rGFAT1 - - WT H577A
j
P<0.0001
GFAT1 activity (%)
GST-GFAT1 WT H577A
k
rGFAT1 WT H577A WT H577A
sg-GFAT1
Ex-GlcN
-100 kDa
-75 kDa
-55 kDa
-45 kDa
-35 kDa
WB: O-GlcNAc
-45 kDa
WB: actin
l
rGFAT1 WT H577A WT H577A
sg-GFAT1
Ex-GlcN
WB: p-p38
WB : p38
WB: actin
n
rGFAT1-H577A
Ex- Glu (mM) 0 3 5 10
Glc- (h) 0 6 12 0 6 12 0 6 12 0 6 12
Fold: 1 3.2 0.9 0.8 3.5 3.1 1.2 4.1 4.3 0.9 4 4.3
WB: p-p38
WB : p38
WB: actin
m
rGFAT1-WT
Glutamate (pmol per gram)
Glc-(h) 0 6 12 0 6 12 0 6 12 0 6 12
Ex-Glu(mM) 0 3 5 10
rGFAT1-H577A
Glutamate (pmol per gram)
Glc-(h) 0 6 12 0 6 12 0 6 12 0 6 12
Ex-Glu(mM) 0 3 5 10
o
P=0.7384
P=0.7592
Glutamate (pmol per gram)
Glc
sg-GFAT1
P=0.4809
P=0.7057
Glutamine (pmol per gram)
Glc
sg-GFAT1

S2

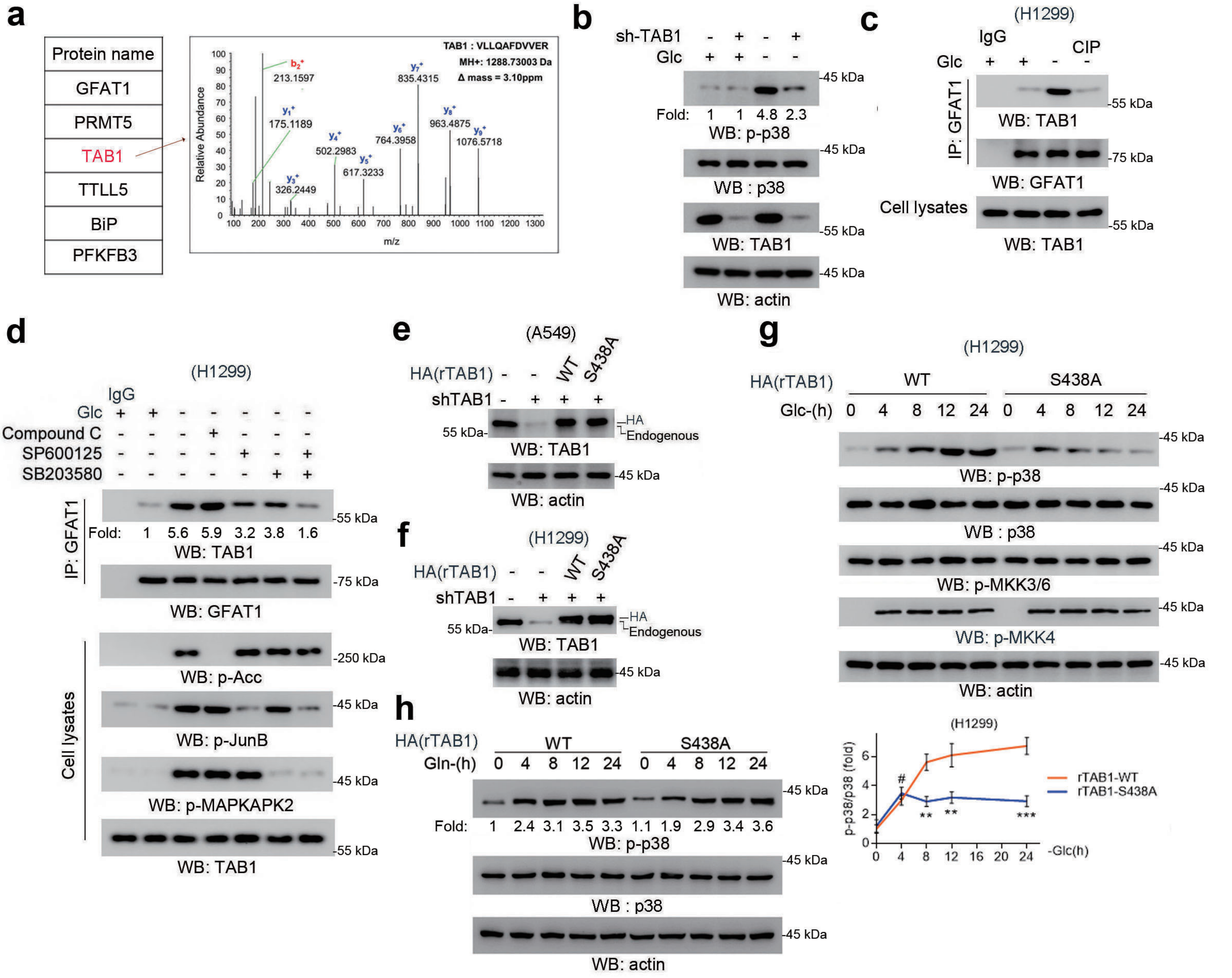

a
Protein name
GFAT1
PRMT5
TAB1
TTLL5
BiP
PFKFB3
TAB1 : VLLQAFDVVER
MH+: 1288.73003 Da
Δ mass = 3.10ppm
Relative Abundance
m/z
213.1597
175.1189
326.2449
502.2983
617.3233
764.3958
835.4315
963.4875
1076.5718
b
sh-TAB1
Glc
Fold: 1 1 4.8 2.3
WB: p-p38
WB : p38
WB: TAB1
WB: actin
c
(H1299)
IgG
CIP
Glc
IP: GFAT1
WB: TAB1
WB: GFAT1
Cell lysates
WB: TAB1
d
(H1299)
IgG
Glc
Compound C
SP600125
SB203580
IP: GFAT1
Fold: 1 5.6 5.9 3.2 3.8 1.6
WB: TAB1
WB: GFAT1
Cell lysates
WB: p-Acc
WB: p-JunB
WB: p-MAPKAPK2
WB: TAB1
e
(A549)
HA(rTAB1) - - WT S438A
shTAB1 - + + +
HA
Endogenous
WB: TAB1
WB: actin
f
(H1299)
HA(rTAB1) - - WT S438A
shTAB1 - + + +
HA
Endogenous
WB: TAB1
WB: actin
g
(H1299)
HA(rTAB1) WT S438A
Glc-(h) 0 4 8 12 24 0 4 8 12 24
WB: p-p38
WB : p38
WB: p-MKK3/6
WB: p-MKK4
WB: actin
(H1299)
p-p38/p38 (fold)
-Glc(h)
rTAB1-WT
rTAB1-S438A
h
HA(rTAB1) WT S438A
Gln-(h) 0 4 8 12 24 0 4 8 12 24
Fold: 1 2.4 3.1 3.5 3.3 1.1 1.9 2.9 3.4 3.6
WB: p-p38
WB : p38
WB: actin

S3

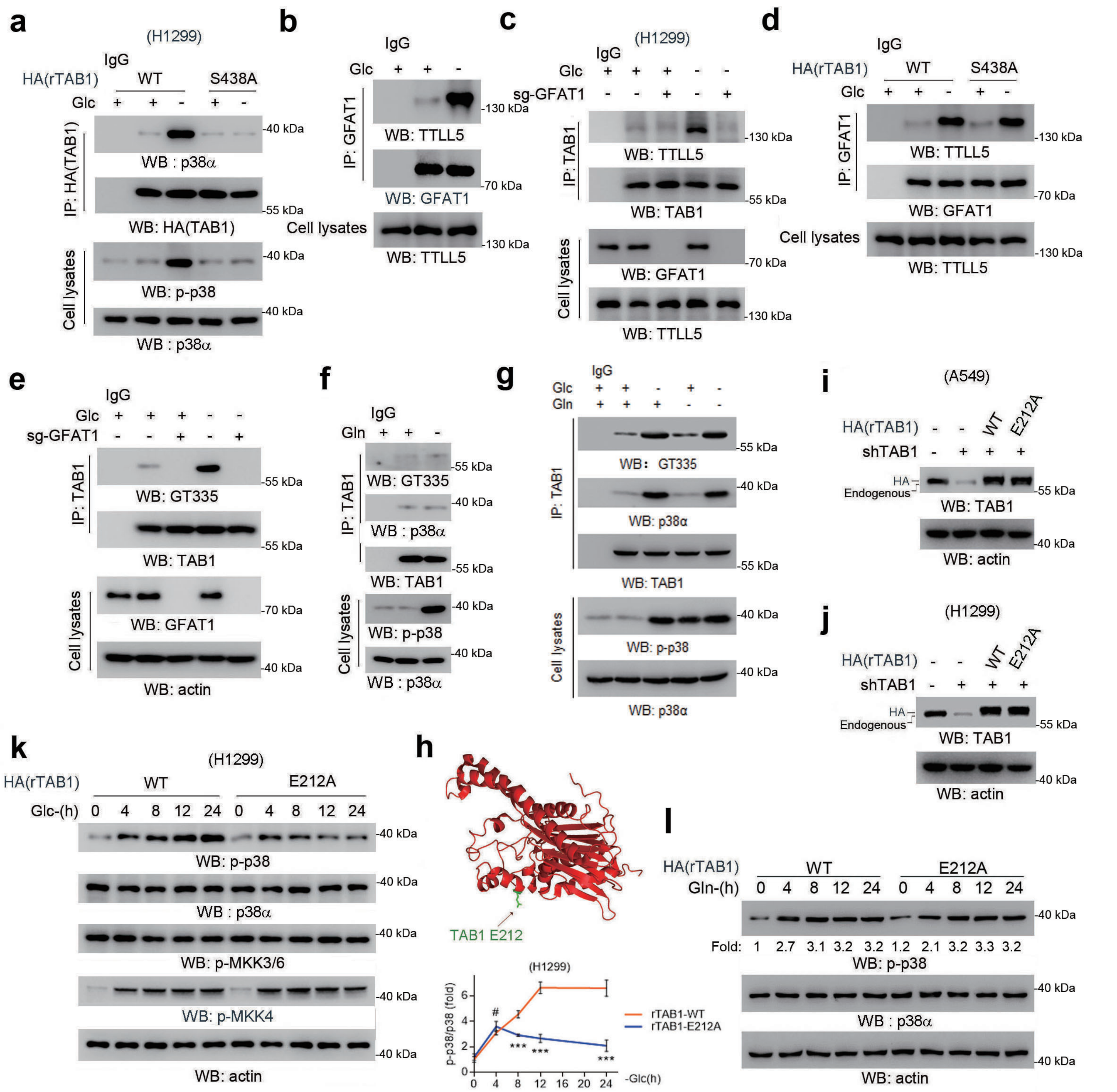
a
(H1299)
IgG
HA(rTAB1) WT S438A
Glc + + - + -
IP: HA(TAB1)
WB : p38α
WB: HA(TAB1)
Cell lysates
WB: p-p38
WB : p38α
-40 kDa
-55 kDa
b
IgG
Glc + + -
IP: GFAT1
WB: TTLL5
WB: GFAT1
Cell lysates
-130 kDa
-70 kDa
c
(H1299)
IgG
Glc + + + - -
sg-GFAT1 - - + - +
IP: TAB1
WB: TTLL5
WB: TAB1
Cell lysates
WB: GFAT1
WB: TTLL5
d
IgG
HA(rTAB1) WT S438A
Glc + + - + -
IP: GFAT1
WB: TTLL5
WB: GFAT1
Cell lysates
WB: TTLL5
e
IgG
Glc + + + - -
sg-GFAT1 - - + - +
IP: TAB1
WB: GT335
WB: TAB1
Cell lysates
WB: GFAT1
WB: actin
f
IgG
Gln + + -
IP: TAB1
WB: GT335
WB : p38α
WB: TAB1
Cell lysates
WB: p-p38
WB : p38α
g
IgG
Glc + + - + -
Gln + + + - -
IP: TAB1
WB: GT335
WB: p38α
WB: TAB1
Cell lysates
WB: p-p38
WB: p38α
i
(A549)
HA(rTAB1) - - WT E212A
shTAB1 - + + +
HA
Endogenous
WB: TAB1
WB: actin
j
(H1299)
HA(rTAB1) - - WT E212A
shTAB1 - + + +
HA
Endogenous
WB: TAB1
WB: actin
k
(H1299)
HA(rTAB1) WT E212A
Glc-(h) 0 4 8 12 24 0 4 8 12 24
WB: p-p38
WB : p38α
WB: p-MKK3/6
WB: p-MKK4
WB: actin
h
TAB1 E212
(H1299)
p-p38/p38 (fold)
-Glc(h)
rTAB1-WT
rTAB1-E212A
l
HA(rTAB1) WT E212A
Gln-(h) 0 4 8 12 24 0 4 8 12 24
Fold: 1 2.7 3.1 3.2 3.2 1.2 2.1 3.2 3.3 3.2
WB: p-p38
WB : p38α
WB: actin

S4

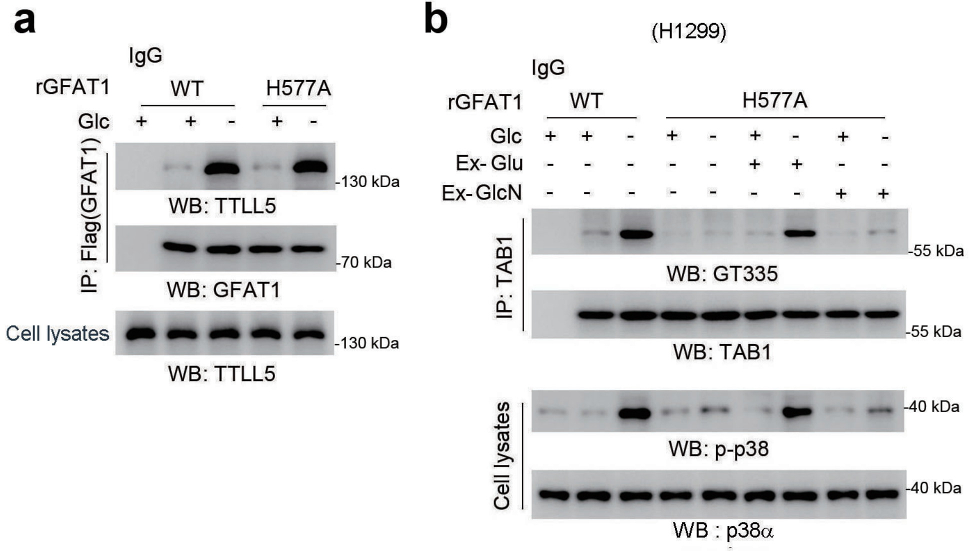
a
IgG
rGFAT1
WT
H577A
Glc
+
+
-
+
-
IP: Flag(GFAT1)
-130 kDa
WB: TTLL5
-70 kDa
WB: GFAT1
Cell lysates
-130 kDa
WB: TTLL5
b
(H1299)
IgG
rGFAT1
WT
H577A
Glc
Ex- Glu
Ex- GlcN
IP: TAB1
-55 kDa
WB: GT335
-55 kDa
WB: TAB1
Cell lysates
-40 kDa
WB: p-p38
-40 kDa
WB : p38α

S5

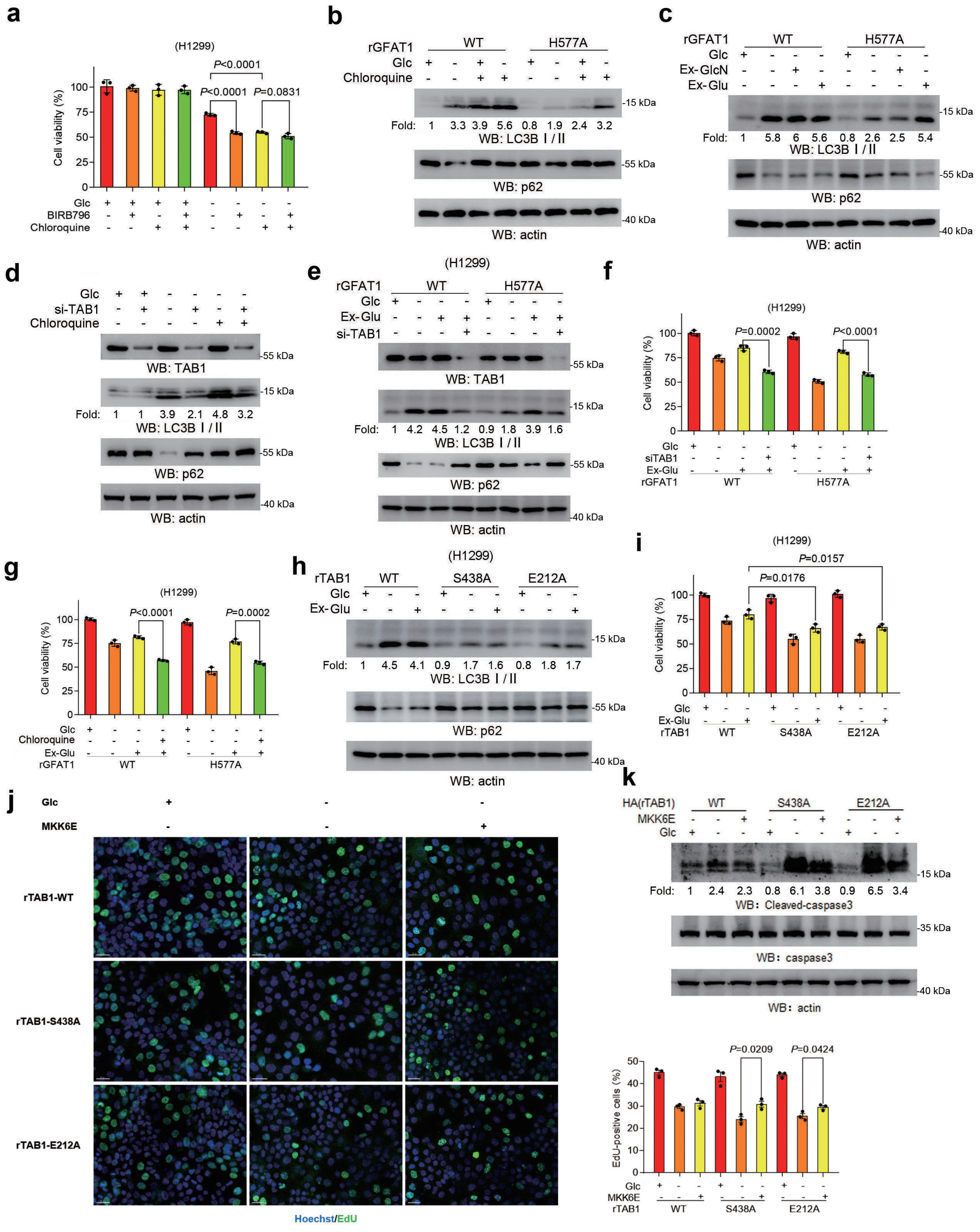
a
(H1299)
P<0.0001
P<0.0001
P=0.0831
Cell viability (%)
Glc
BIRB796
Chloroquine
b
rGFAT1
WT
H577A
Glc
Chloroquine
-15 kDa
Fold: 1 3.3 3.9 5.6 0.8 1.9 2.4 3.2
WB: LC3B Ⅰ/Ⅱ
-55 kDa
WB: p62
-40 kDa
WB: actin
c
rGFAT1
WT
H577A
Glc
Ex-GlcN
Ex-Glu
-15 kDa
Fold: 1 5.8 6 5.6 0.8 2.6 2.5 5.4
WB: LC3B Ⅰ/Ⅱ
-55 kDa
WB: p62
-40 kDa
WB: actin
d
Glc
si-TAB1
Chloroquine
-55 kDa
WB: TAB1
-15 kDa
Fold: 1 1 3.9 2.1 4.8 3.2
WB: LC3B Ⅰ/Ⅱ
-55 kDa
WB: p62
-40 kDa
WB: actin
e
(H1299)
rGFAT1
WT
H577A
Glc
Ex-Glu
si-TAB1
-55 kDa
WB: TAB1
-15 kDa
Fold: 1 4.2 4.5 1.2 0.9 1.8 3.9 1.6
WB: LC3B Ⅰ/Ⅱ
-55 kDa
WB: p62
-40 kDa
WB: actin
f
(H1299)
P=0.0002
P<0.0001
Cell viability (%)
Glc
siTAB1
Ex-Glu
rGFAT1
WT
H577A
g
(H1299)
P<0.0001
P=0.0002
Cell viability (%)
Glc
Chloroquine
Ex-Glu
rGFAT1
WT
H577A
h
(H1299)
rTAB1
WT
S438A
E212A
Glc
Ex-Glu
-15 kDa
Fold: 1 4.5 4.1 0.9 1.7 1.6 0.8 1.8 1.7
WB: LC3B Ⅰ/Ⅱ
-55 kDa
WB: p62
-40 kDa
WB: actin
i
(H1299)
P=0.0157
P=0.0176
Cell viability (%)
Glc
Ex-Glu
rTAB1
WT
S438A
E212A
j
Glc
MKK6E
rTAB1-WT
rTAB1-S438A
rTAB1-E212A
Hoechst/EdU
k
HA(rTAB1)
WT
S438A
E212A
MKK6E
Glc
-15 kDa
Fold: 1 2.4 2.3 0.8 6.1 3.8 0.9 6.5 3.4
WB：Cleaved-caspase3
-35 kDa
WB：caspase3
-40 kDa
WB：actin
P=0.0209
P=0.0424
EdU-positive cells (%)
Glc
MKK6E
rTAB1
WT
S438A
E212A

S6

a

TAB pS438 | TAB pS438+ Block peptide

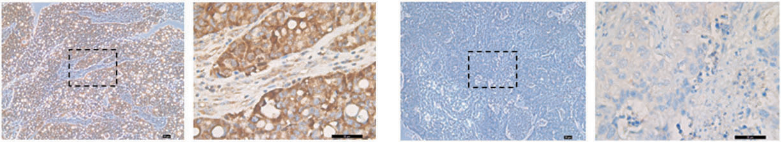

b

TAB1 pSer438 \ GFAT1		Low			High		
		0	1	2	3	4	6
Low	0	0	0	0	0	0	0
	1	0	6	8	1	6	1
	2	0	0	4	1	3	0
High	3	1	1	4	1	14	4
	4	0	0	2	3	11	1
	6	0	0	1	1	8	5

Person correlation: r=0.458; *P*<0.001

c

p-p38 \ GFAT1		Low			High		
		0	1	2	3	4	6
Low	0	0	0	0	0	0	0
	1	0	7	3	0	7	0
	2	0	0	6	0	3	2
High	3	1	0	8	3	15	3
	4	0	0	1	1	14	1
	6	0	0	1	3	3	5

Person correlation: r=0.421; *P*<0.001

Supplementary Figure S1. GFAT1 activates p38 MAPK and promotes cell survival and tumorigenesis through its enzymatic activity.

a Glucose deprivation, but not glutamine deprivation, inhibited cell survival in GFAT1-deleted cells. H1299 cells with or without deleted GFAT1 were cultured for 36 h under glucose or glutamine deprivation. Cellular viability was examined by CCK8 assay. **b**, **c** Extended glucose or glutamine deprivation regulated GFAT1 expression. A549 cells were cultured for indicated time under glucose deprivation (**b**) or glutamine deprivation (**c**). Immunoblotting analyses were performed using the indicated antibodies. **d** GFAT1 deletion inhibited p38 activation at late time points. H1299 cells with or without deleted GFAT1 were cultured for indicated time under glucose deprivation. Immunoblotting analyses were performed using the indicated antibodies. **e** p38 suppression impaired cell survival. H1299 cells were pretreated with or without BIRB796 (1μM) for 1 h before being cultured under glucose deprivation. Cellular viability was examined by CCK8 assay at 36 h post glucose deprivation treatment. Immunoblotting analyses were performed using the indicated antibodies. **f** Expression of MKK6E rescued p38 activation in GFAT1-deleted cells. A549 cells with or without deleted GFAT1 and expression of MKK6E were cultured for 8h under glucose deprivation. Immunoblotting analyses were performed using the indicated antibodies. **g**, **h** A549 (**g**) and H1299 (**h**) cells with deleted GFAT1 were reconstituted with expression of WT rGFAT1 or rGFAT1 H577A. Immunoblotting analyses were performed using the indicated antibodies. **i** Expression of rGFAT1-H577A inhibited cell survival. H1299 cells with deleted GFAT1 and reconstituted expression of WT rGFAT1 or rGFAT1-H577A were cultured under

glucose deprivation. Cellular viability was examined by CCK8 assay at 36 h post glucose deprivation treatment. **j** Enzyme activities of WT GFAT1 and GFAT1-H577A were measured. **k** Exogenous glucosamine rescued overall O-GlcNAcylation in GFAT1-deleted and rGFAT1-H577A-expressing cells. A549 cells with deleted GFAT1 and reconstituted expression of WT rGFAT1 or rGFAT1-H577A were added with exogenous glucosamine (Ex-GlcN). Immunoblotting analyses were performed using the indicated antibodies. **l** Exogenous glucosamine failed to rescue p38 activation in GFAT1-deleted and rGFAT1-H577A-expressing cells. A549 cells with deleted GFAT1 and reconstituted expression of WT rGFAT1 or rGFAT1-H577A were cultured for 8 h under glucose deprivation and were added with exogenous glucosamine (Ex-GlcN). Immunoblotting analyses were performed using the indicated antibodies. **m**, **n** Addition of exogenous glutamate increased intracellular glutamate concentrations and rescued p38 activation in rGFAT1-H577A-expressing cells. A549 cells with deleted GFAT1 and reconstituted expression of WT rGFAT1 or rGFAT1-H577A were cultured for indicated time under glucose deprivation with exogenous glutamate (Ex-Glu), intracellular glutamate concentrations were measured (**m**). Immunoblotting analyses were performed using the indicated antibodies (**n**). **o** GFAT1 deletion did not reduce intracellular glutamate and glutamine concentrations. A549 cells with deleted GFAT1 were cultured for 8 h under glucose deprivation, intracellular glutamate and glutamine concentrations were measured. In **a**, **d**, **e**, **i**, **j** **m** and **o**, the values are presented as mean ± SEM, n=3; P values (#$P > 0.05$, ***$P < 0.001$, Student's t-test, two-sided) with control or the indicated groups are presented.

Supplementary Figure S2. TAB1 interacts with GFAT1 in a Ser438 phosphorylation-dependent manner.

a Representative GFAT1-assoicated proteins identified by mass spectrometry were shown. A fragment of TAB1 was detected. A549 cells with stable expression of Flag-GFAT1 were cultured for 8 h under glucose deprivation. Cellular extracts subjected to immunoprecipitation with an anti-Flag antibody were analyzed by mass spectrometry. **b** TAB1 depletion inhibited p38 activation. A549 cells with depleted TAB1 were cultured for 8 h under glucose deprivation. Immunoblotting analyses were performed using the indicated antibodies. **c** TAB1–GFAT1 interaction was phosphorylation-dependent. H1299 cells were cultured for 8 h under glucose deprivation. Immunoprecipitation and immunoblotting analyses were performed using the indicated antibodies. **d** JNK/p38 mediated TAB1–GFAT1 interaction. H1299 cells were pretreated with Compound C (10 μM), SP600125 (20 μM), and SB203580 (10 μM) for 1 h before being cultured for 8h under glucose deprivation. Immunoprecipitation and immunoblotting analyses were performed using the indicated antibodies. **e**, **f** A549 (**e**) and H1299 (**f**) cells with depleted TAB1 were reconstituted with expression of WT rTAB1 or rTAB1-S438A. **g** Expression of rTAB1-S438A inhibited p38 activation at late time points. H1299 cells with depleted TAB1 and reconstituted expression of WT rTAB1 or rTAB1-S438A were cultured for indicated time under glucose deprivation. Immunoblotting analyses were performed using the indicated antibodies. **h** Glutamine deprivation failed to inhibit p38 activation in rTAB1-S438A-expressing cells. A549

cells with depleted TAB1 and reconstituted expression of WT rTAB1 or rTAB1-S438A were cultured for indicated time under glutamine deprivation. Immunoblotting analyses were performed using the indicated antibodies. In **g**, the values are presented as mean ± SEM, n=3; #$P > 0.05$, **$P < 0.01$, ***$P < 0.001$, Student's *t*-test, two-sided.

Supplementary Figure S3. TAB1–GFAT1–TTLL5 complex promotes TAB1 glutamylation and p38 MAPK activation.

a Expression of rTAB1-S438A inhibited TAB1–p38 interaction. H1299 cells with depleted TAB1 and reconstituted expression of WT rTAB1 or rTAB1-S438A were cultured for 8 h under glucose deprivation. Immunoprecipitation and immunoblotting analyses were performed using the indicated antibodies. **b** Glucose deprivation promoted GFAT1–TTLL5 interaction. A549 cells were cultured for 8h under glucose deprivation. Immunoprecipitation and immunoblotting analyses were performed using the indicated antibodies. **c** GFAT1 mediated TAB1–GFAT1–TTLL5 complex formation. H1299 cells with or without deleted GFAT1 were cultured for 8 h under glucose deprivation. Immunoprecipitation and immunoblotting analyses were performed using the indicated antibodies. **d** Expression of rTAB1-S438A did not reduce GFAT1–TTLL5 interaction. A549 cells with depleted TAB1 and reconstituted expression of WT rTAB1 or rTAB1-S438A were cultured for 8 h under glucose deprivation. Immunoprecipitation and immunoblotting analyses were performed using the indicated antibodies. **e** GFAT1 deletion inhibited TAB1 glutamylation. A549 cells with or without deleted GFAT1 were cultured for 8 h under glucose deprivation. Immunoprecipitation and immunoblotting

analyses were performed using the indicated antibodies. **f**, **g** Glutamine deprivation alone failed to regulate TAB1 glutamylation. A549 cells were cultured for 8 h under glutamine deprivation (**f**) or simultaneous glucose/glutamine deprivation (**g**). Immunoprecipitation and immunoblotting analyses were performed using the indicated antibodies. **h** A structure image of TAB1; TAB1 Glu212 is colored green. **i**, **j** A549 (**i**) and H1299 (**j**) cells with depleted TAB1 were reconstituted with expression of WT rTAB1 or rTAB1-E212A. Immunoblotting analyses were performed using the indicated antibodies. **k** Expression of rTAB1-E212A inhibited p38 activation at late time points. H1299 cells with depleted TAB1 and reconstituted expression of WT rTAB1 or rTAB1-E212A were cultured for indicated time under glucose deprivation. Immunoblotting analyses were performed using the indicated antibodies. **l** Glutamine deprivation failed to inhibit p38 activation in rTAB1-E212A-expressing cells. A549 cells with depleted TAB1 and reconstituted expression of WT rTAB1 or rTAB1-E212A were cultured for indicated time under glutamine deprivation. Immunoblotting analyses were performed using the indicated antibodies. In **k**, the values are presented as mean ± SEM, n=3; #*P* > 0.05, ****P* < 0.001, Student's *t*-test, two-sided.

Supplementary Figure S4. Glutamate derived from GFAT1 is required for TAB1 glutamylation.

a Expression of rGFAT1-H577A did not reduce GFAT1–TTLL5 interaction. A549 cells with deleted GFAT1 and reconstituted expression of WT rGFAT1 or rGFAT1-H577A were cultured for 8 h under glucose deprivation. Immunoprecipitation and

immunoblotting analyses were performed using the indicated antibodies. **b** Exogenous glutamate rescued TAB1 glutamylation in rGFAT1-H577A-expressing cells. H1299 cells with deleted GFAT1 and reconstituted expression of WT rGFAT1 or rGFAT1-H577A were cultured for 8h under glucose deprivation with exogenous glutamate (Ex-Glu) or glucosamine (Ex-GlcN). Immunoprecipitation and immunoblotting analyses were performed using the indicated antibodies.

Supplementary Figure S5. p38 MAPK activation is critical for autophagy and cell survival.

a p38 or autophagy inhibition suppressed cell survival. H1299 cells were pretreated with BIRB796 (20 μM) and chloroquine (100 μM) for 1 h before being cultured under glucose deprivation. Cellular viability was examined by CCK8 assay at 36 h post glucose deprivation treatment. **b** Expression of rGFAT1-H577A inhibited autophagy. A549 cells with deleted GFAT1 and reconstituted expression of WT rGFAT1 or rGFAT1-H577A were pretreated with BIRB796 (20 μM) for 1 h before being cultured for 8h under glucose deprivation. Immunoblotting analyses were performed using the indicated antibodies. **c** Exogenous glutamate rescued autophagy in rGFAT1-H577A-expressing cells. A549 cells with deleted GFAT1 and reconstituted expression of WT rGFAT1 or rGFAT1-H577A were cultured for 8 h under glucose deprivation and were added with exogenous glutamate (Ex-Glu) or glucosamine (Ex-GlcN). Immunoblotting analyses were performed using the indicated antibodies. **d** TAB1 depletion inhibited autophagy. A549 cells were transfected with TAB1 siRNA and were pretreated with

chloroquine (100 μM) for 1 h before being cultured for 8 h under glucose deprivation. Immunoblotting analyses were performed using the indicated antibodies. **e**, **f** Exogenous glutamate rescued autophagy and cell survival in rGFAT1-H577A-expressing cells, but not when TAB1 was depleted. H1299 cells with deleted GFAT1 and reconstituted expression of WT rGFAT1 or rGFAT1-H577A were transfected with TAB1 siRNA and were cultured under glucose deprivation with exogenous glutamate (Ex-Glu). Immunoblotting analyses were performed using the indicated antibodies (**e**). Cellular viability was examined by CCK8 assay at 36 h post glucose deprivation treatment (**f**). **g** Autophagy inhibition suppressed cell survival rescued by exogenous glutamate in rGFAT1-H577A-expressing cells. H1299 cells with deleted GFAT1 and reconstituted expression of WT rGFAT1 or rGFAT1-H577A were pretreated with chloroquine (100 μM) for 1 h before being cultured under glucose deprivation and added with exogenous glutamate (Ex-Glu). Cellular viability was examined by CCK8 assay at 36 h post glucose deprivation treatment. **h**, **i** Exogenous glutamate failed to rescue autophagy and cell survival in rTAB1-S438A- and rTAB1-E212A-expressing cells. H1299 cells with depleted TAB1 and reconstituted expression of WT rTAB1, rTAB1-S438A or rTAB1-E212A were cultured under glucose deprivation with exogenous glutamate (Ex-Glu). Immunoblotting analyses were performed using the indicated antibodies (**h**). Cellular viability was examined by CCK8 assay at 36 h post glucose deprivation treatment (**i**). **j**, **k** Expression of MKK6E rescued cell proliferation and suppressed apoptosis in rTAB1-S438A- and rTAB1-E212A-expressing cells. A549 cells with depleted TAB1 and reconstituted expression of WT rTAB1, rTAB1-S438A

or rTAB1-E212A were cultured under glucose deprivation, EdU labeling staining was performed (**j**). Immunoblotting analyses were performed using the indicated antibodies (**k**). Scale bar, 30 μm. In **a**, **f**, **g**, **i** and **j**), the values are presented as mean ± SEM, n=3; *P* values (Student's *t*-test, two-sided) with control or the indicated groups are presented.

Supplementary Figure S6. GFAT1 is related to TAB1 S438 phosphorylation and p38 activation.

a The TAB1 pS438 antibody specificity was validated using IHC analyses with specific peptide that blocks TAB1 S438 phosphorylation. Scale bar, 50 μm. **b** Semi-quantitative scoring and correlation analysis indicating the correlation between GFAT1 and TAB1 pSer438 (Person correlation test; r = 0.458, *P* < 0.001). **c** Semi-quantitative scoring and correlation analysis indicating the correlation between GFAT1 and p-p38 (Person correlation test; r = 0.421, *P* < 0.001).
